# Expanded expression of pro-neurogenic factor SoxB1 during larval development of gastropod *Lymnaea stagnalis* suggests preadaptation to prolonged neurogenesis in Mollusca

**DOI:** 10.3389/fnins.2024.1346610

**Published:** 2024-04-04

**Authors:** Anastasia I. Kurtova, Alexander D. Finoshin, Margarita S. Aparina, Guzel R. Gazizova, Olga S. Kozlova, Svetlana N. Voronova, Elena I. Shagimardanova, Evgeny G. Ivashkin, Elena E. Voronezhskaya

**Affiliations:** ^1^Koltsov Institute of Developmental Biology, Russian Academy of Sciences, Moscow, Russia; ^2^Severtsov Institute of Ecology and Evolution, Russian Academy of Sciences, Moscow, Russia; ^3^Regulatory Genomics Research Center, Institute of Fundamental Medicine and Biology, Kazan Federal University, Kazan, Russia; ^4^Life Improvement by Future Technologies Center “LIFT”, Moscow, Russia; ^5^Skolkovo Institute of Science and Technology, Moscow, Russia

**Keywords:** *Lymnaea stagnalis*, neurogenesis, SoxB1, SoxB2, gastropod mollusks, ganglia formation, trochophore, veliger

## Abstract

**Introduction:**

The remarkable diversity observed in the structure and development of the molluscan nervous system raises intriguing questions regarding the molecular mechanisms underlying neurogenesis in *Mollusca*. The expression of SoxB family transcription factors plays a pivotal role in neuronal development, thereby offering valuable insights into the strategies of neurogenesis.

**Methods:**

In this study, we conducted gene expression analysis focusing on SoxB-family transcription factors during early neurogenesis in the gastropod *Lymnaea stagnalis*. We employed a combination of hybridization chain reaction in situ hybridization (HCR-ISH), immunocytochemistry, confocal microscopy, and cell proliferation assays to investigate the spatial and temporal expression patterns of *LsSoxB1* and *LsSoxB2* from the gastrula stage to hatching, with particular attention to the formation of central ring ganglia.

**Results:**

Our investigation reveals that *LsSoxB1* demonstrates expanded ectodermal expression from the gastrula to the hatching stage, whereas expression of *LsSoxB2* in the ectoderm ceases by the veliger stage. *LsSoxB1* is expressed in the ectoderm of the head, foot, and visceral complex, as well as in forming ganglia and sensory cells. Conversely, *LsSoxB2* is mostly restricted to the subepithelial layer and forming ganglia cells during metamorphosis. Proliferation assays indicate a uniform distribution of dividing cells in the ectoderm across all developmental stages, suggesting the absence of distinct neurogenic zones with increased proliferation in gastropods.

**Discussion:**

Our findings reveal a spatially and temporally extended pattern of SoxB1 expression in a gastropod representative compared to other lophotrochozoan species. This prolonged and widespread expression of SoxB genes may be interpreted as a form of transcriptional neoteny, representing a preadaptation to prolonged neurogenesis. Consequently, it could contribute to the diversification of nervous systems in gastropods and lead to an increase in the complexity of the central nervous system in *Mollusca*.

## 1 Introduction

Mollusca, a highly diverse phylum within Bilateria, exhibits an astonishing array of body forms, lifestyles, and ecological adaptations. This remarkable diversity is not only visible in their external morphology but is also deeply intertwined with the developmental patterns of the nervous system of these organisms. The structure of the molluscan nervous system reflects their lifestyles and demonstrates high plasticity at the morphological level, from the scattered ganglia in Bivalves to the highly centralized brain of Cephalopods (Bullock and Horridge, 1965; Schmidt-Rhaesa et al., 2015). It is an intriguing question how such a diversity of nervous systems arises during development and emerges in evolution.

Our knowledge about the formation of the nervous system in mollusks is mostly restricted to the appearance of already differentiated neurons (Croll, 2009; Nielsen, 2012; Richter et al., 2015; Voronezhskaya and Croll, 2015). Commonly used markers like serotonin, catecholamines, and FMRFamide-related peptides have proven effective in visualizing specific neuronal subsets across various invertebrate groups (Schmidt-Rhaesa et al., 2015). However, it is imperative to emphasize that, whether employed individually or collectively, these markers do not provide a comprehensive visualization of the entire nervous system in mollusks because the pan-neuronal marker is still lacking. Contrary to polychaete larvae, acetylated and tyrosinated alpha-tubulin mark the ciliary structures only and not the nerve elements in representative molluscan larvae (Croll, 2000, 2009; Kristof et al., 2016; Battonyai et al., 2018; Pavlicek et al., 2018; Yurchenko et al., 2018). Moreover, the spatial and temporal distribution of neurons expressing specific transmitter phenotypes varies significantly between molluscan classes and even within one family, making it difficult for comparative analysis of neurogenesis (Sakharov, 1976; Croll, 2000; Moroz, 2009, 2021). Based on the existing data for other invertebrate groups, it seems reasonable to look at the early neurogenic events. Particularly, the data about neurogenic stem cells and expression of transcriptional factors that precede neuron specification. Such factors have been identified for all the main groups of Eumetazoa representatives from cnidarians to vertebrates and demonstrated as evolutionarily highly conserved features (Marlow et al., 2014; Moroz, 2015; Arendt et al., 2016; Arendt, 2018; Martín-Durán et al., 2018). In the case of mollusks, such sets of early and late neurogenic factors have been described in detail only for cephalopods (Focareta and Cole, 2016; Deryckere et al., 2021; Duruz et al., 2023), which demonstrate a lot of specific features in their complex nervous system. Data about the presence and distribution of pan-neuronal proneurogenic and neurogenic factors are scarce in the case of other Molluscan groups, particularly in gastropods.

Sox genes, characterized by the presence of the high mobility group (HMG) DNA binding domain, constitute a group of transcription factors with pivotal roles in cell specification and tissue differentiation (Pevny and Placzek, 2005). Among the panoply of Sox genes, it is the SoxB representatives that emerge as key players in neuronal development processes. Their early expression during gastrulation contributes substantially to ectodermal patterning and gastrulation movements (Okuda et al., 2010). During neurogenesis of vertebrates, SoxB1 and SoxB2 family genes maintain the accurate balance between cell proliferation and differentiation, acting as a gatekeeper to inhibit premature differentiation (Bylund et al., 2003; Masui et al., 2007). Moreover, SoxB1 genes play a pivotal role in neural subtype differentiation within the central nervous system, underlining their significance in the intricate process of neural specification (Pevny and Placzek, 2005; Kiefer, 2007; Panayi et al., 2010). SoxB1 expression occurs in neurogenic zones where it maintains the cells’ ability to proliferate and inhibits further differentiation (Bylund et al., 2003; Masui et al., 2007). In turn, SoxB2 group genes repress SoxB1 activity and allow progenitor cells to differentiate into neurons (Pevny and Placzek, 2005; Kiefer, 2007).

Sox genes have been identified in many invertebrate species (Phochanukul and Russell, 2010). In addition to conservative features, the specific role of SoxB1 and SoxB2 in larval development and neurogenesis has been mentioned in different groups. In some cnidarians, SoxB genes act as one of the key regulators of larval morphogenesis (Chrysostomou et al., 2022). In the nematode *C. elegans*, SoxB1 and SoxB2 are largely recruited into the mechanism of the larval to adult transition of the nervous system (Vidal et al., 2015). SoxB expression has also been detected in cnidarians (Magie et al., 2005; Shinzato et al., 2008; Richards and Rentzsch, 2014), flatworms (Dong et al., 2014; Monjo and Romero, 2015), acoels (Semmler et al., 2010), annelids (Kerner et al., 2009; Sur et al., 2020), insects (Buescher et al., 2002; Wilson and Dearden, 2008), and bryozoans (Fuchs et al., 2011).

Despite the emerging understanding of Sox gene functions in various organisms, the specifics of their expression and role remain largely unexplored outside of well-studied models such as *Drosophila*, sea urchins, and nematodes. In particular, their functions in the most diverse group of Lophotrochozoans – Mollusca – remain obscure. Recent studies on cephalopods have shed light on SoxB-family gene expression and their involvement in neuronal precursor specification within the head ectoderm and developing ganglia (Focareta and Cole, 2016; Deryckere et al., 2021; Duruz et al., 2023). However, cephalopods have a largely modified development without larvae in their life cycle. Information regarding SoxB gene expression in basal molluscan groups possessing true larvae as well as its correlation with larval neurogenesis is scarce (Le Gouar et al., 2004; Huan et al., 2020; Tan et al., 2022).

We use larvae of the freshwater gastropod *Lymnaea stagnalis* (*L. stagnalis*) to study the expression patterns of SoxB1 and SoxB2 in the course of development from gastrulation to hatching. In addition, we apply FMRFamide immunostaining to visualize specific neuronal elements. Immunostaining using FMRFamide, serotonin, and tubulin antibodies is widely used to reveal the nervous system in developing larvae in a variety of invertebrates (Schmidt-Rhaesa et al., 2015). In the case of *L. stagnalis*, any isoforms of tubulin mark the ciliary structures only and not the neuronal processes (own data). Serotonin-positive cells appear late in neurogenesis and are restricted to the anterior ganglia in *L. stagnalis* (Marois and Croll, 1992). To the contrary, FMRFamide-positive cells appear as the earliest nerve elements, located both in ganglia and in the periphery and mark the neuropile of all ganglia as well (Croll and Voronezhskaya, 1996; Voronezhskaya and Elekes, 1996; Voronezhskaya and Croll, 2015; Nezlin and Voronezhskaya, 2017). Parallel visualization of FMRFamide-immunoreactive elements with Sox gene expression allows us to correlate the location of presumptive neurogenic areas with the emerging larval nervous system.

Employing modern techniques such as mRNA *in situ* hybridization chain reaction (HCR-ISH), immunohistochemistry (IHC) for SoxB1 and FMRFamide, and proliferation assays, our research provides a comprehensive outlook into the correlation between SoxB1\SoxB2-expressing cells, the formation of central ring ganglia, and the presence of active proliferation zones within the larval body. These findings provide crucial insights into the conserved role of SoxB family proteins in neurogenesis across the evolutionary spectrum. Furthermore, they underscore the crucial distinction in the expanded SoxB1 expression that is specific to gastropod mollusks. This study thus contributes to our broader understanding of Sox gene functions in neurogenesis in non-model organisms and highlights the importance of such studies in diverse species.

## 2 Results

**Figure 1:**
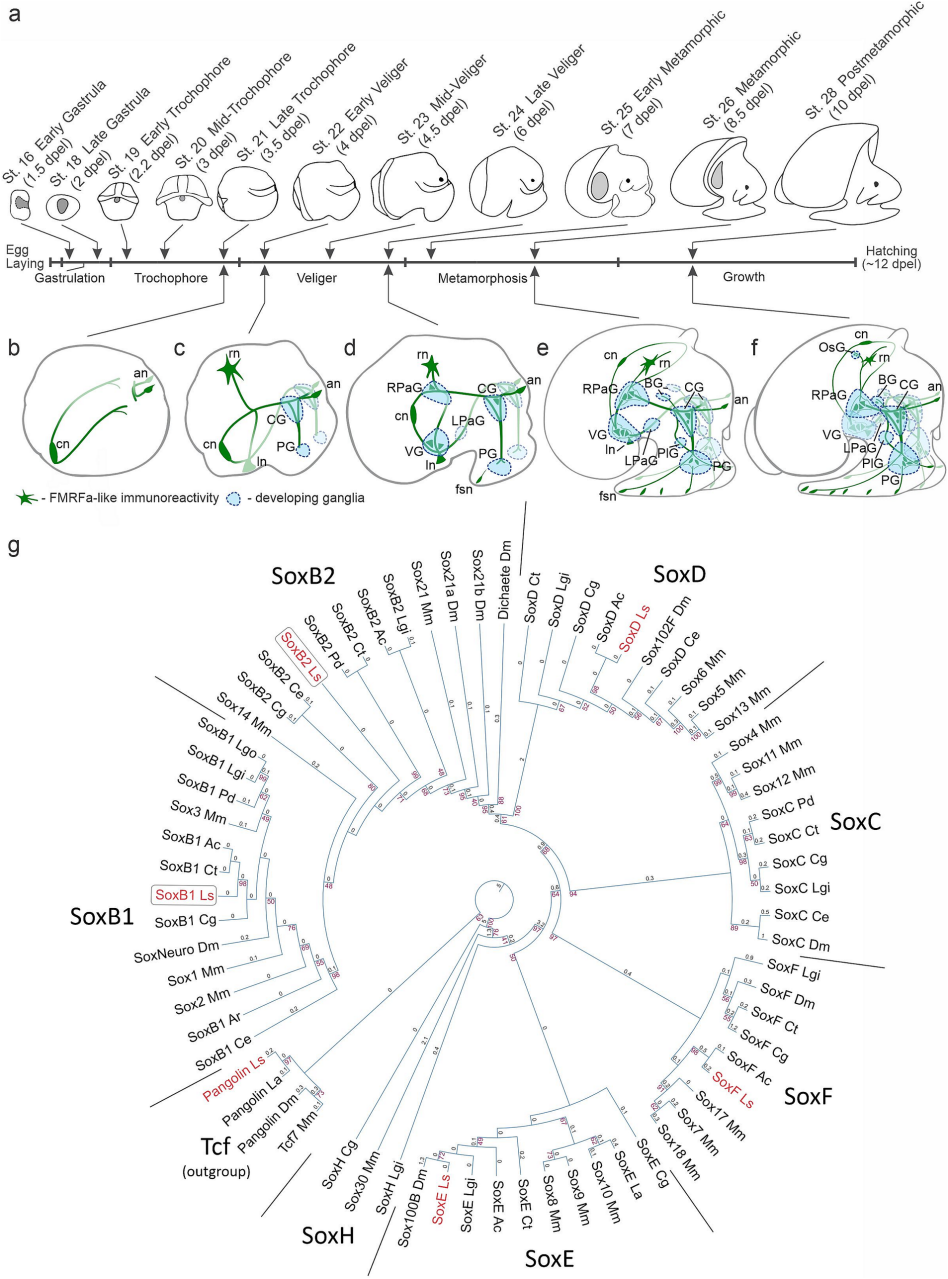
Developmental staging and phylogenetic analysis of Sox genes in *Lymnaea stagnalis*. **(a)** Subsequent developmental stages of *L. stagnalis* and a schematic view of the stages analyzed in the current study [adapted from Meshcheryakov (1990) with modifications]. The timing of development is indicated in days post-egg laying (dpel). **(b–f)** Schematic drawings of the nervous system development based on descriptions by Meshcheryakov (1990) and Croll and Voronezhskaya (2015). **(b)** Symmetrical appearance of early peripheral nerve elements. **(c)** Formation of paired cerebral and pedal ganglia. **(d,e)** Emergence of visceral loop ganglia and torsion. **(f)** Centralization of ganglia to the esophageal ring. For a detailed description of nervous system development, see the text. Green, FMRFamide-like immunoreactive nerve elements; blue, developing ganglia. an, apical neurons; BG, buccal ganglion; CG, cerebral ganglion; cn, caudal peripheral neuron; fsn, foot sensory neuron; ln, left peripheral neuron; LPaG, left parietal ganglion; OsG, osphradial ganglion; PG, pedal ganglion; PlG, pleural ganglion; rn, right peripheral neuron; RPaG, right parietal ganglion; VG, visceral ganglion. **(g)** Maximum likelihood phylogeny of conserved HMG domains (81 aa) from Sox proteins across various species. The red numbers at the nodes indicate bootstrap percentages above 40%. The branch length values are written above the phylogram braces. Detailed species names and sequence accession numbers can be found in the Supplementary Table S1. The tree utilizes Tcf/Pangolin as an outgroup. The HMG domains of recognized Sox families cluster together in the tree as anticipated and are marked as SoxB1, SoxB2, SoxD, SoxC, SoxF, SoxE, and SoxH. *L. stagnalis* genes are in red, while SoxB paralogs are in frames. Species abbreviations: Ac, *Aplysia californica*; Ar, *Acanthochitona rubrolineata*; Ce, *Caenorhabditis elegans*; Cg, *Crassostrea gigas*; Ct, *Capitella teleta*; Dm, *Drosophila melanogaster*; La, *Lingula anatina*; Lgi, *Lottia gigantea*; Lgo, *Lottia goshimai*; Ls, *Lymnaea stagnalis*; Mm, *Mus musculus*; Pd, *Platynereis dumerilii*.

The larval development of *L. stagnalis* occurs within egg capsules deposited in egg masses by mature snails. This developmental process typically spans approximately 12 ± 0.5 days at 25°C. Within a single egg mass, larvae undergo synchronous development across distinct stages, including early cleavage, gastrulation, trochophore, veliger, metamorphosis, postmetamorphic growth, and hatching (Meshcheryakov, 1990; Ivashkin et al., 2015). A schematic overview of the stages examined in this study, along with stage names following the table of *L. stagnalis* normal development by Meshcheryakov (1990), and developmental timing (presented as days post egg laying, dpel), is presented in Figure 1a. Our research specifically focuses on the stages spanning from gastrula to hatchlings, during which the full course neurodevelopment takes place.

In studying the nervous system, we rely on foundational data derived from prior investigations, which establish the use of FMRFamide as the most appropriate neuronal marker for visualizing *L. stagnalis* nervous system developmental dynamics. FMRFamide immunostaining reveals the earliest peripheral cells and their processes scaffolding the neuropile of forming ganglia, and later the neurons within all developing ganglia (Croll and Voronezhskaya, 1996; Voronezhskaya and Elekes, 1996; Nezlin and Voronezhskaya, 2017). Additionally, FMRFamide labels peripheral sensory cells and the local neural networks (Croll, 2000; Faccioni-Heuser et al., 2004; Voronezhskaya and Croll, 2015). Thus, FMRFamide-positive elements provide comprehensive visualization of the *L. stagnalis* nervous system throughout all developmental stages examined.

The formation of the *L. stagnalis* nervous system starts at the trochophore stage with the appearance of early peripheral cells in the posterior (central, left, and right neurons) and anterior (apical neurons) regions of the embryonic body. Processes of early peripheral cells provide scaffolding upon which the central ganglia will subsequently develop (Figures 1b,c). At the veliger stage, the paired symmetrical cerebral (CG) and pedal (PG) ganglia appear at the head and forming foot regions (Figure 1c). By the end of the veliger stage (late veliger), the ventral (VG), right parietal (RPaG), and left parietal (LPaG) ganglia start to form along the visceral loop (Figure 1d). During metamorphosis, the VG shifts ventrally, and the RPaG moves right and dorsally, forming a figure-of-eight pattern. Thus, the crossing of the visceral connectives, the so-called chiastoneury, occurs at the metamorphic stages (Figures 1d,e). Paired buccal ganglia (BG) appear anterio-dorsally to the CG, and pleural ganglia (PlG) form adjacent to the cerebro-pedal connectives (Figure 1e). In the course of further growth, all ganglia of the visceral loop move rostrodorsally, undergo partial detorsion and centralization, and finally locate around the esophagus. The unpaired osphradial ganglion appears at the mantle region (Figure 1f). The anatomical arrangement of the *L. stagnalis* CNS resembles its adult organization by hatching and comprises paired cerebral, pedal, buccal, pleural, and parietal ganglia, and an unpaired visceral and a peripheral osphradial ganglion (Figure 1f). Thus, the appearance and arrangement of the gastropod mollusks ganglionic nervous system are much more complicated and significantly differ from the linear organization characteristic of most other invertebrate groups like annelids, polyplacophoran mollusks, and insects (Croll and Voronezhskaya, 1996; Croll, 2000; Schmidt-Rhaesa et al., 2015). Additionally, *L. stagnalis* features an extensive peripheral nervous system, including a plexus in the head, tentacles, and foot. Notably, the number of peripheral neurons in this plexus exceeds the number of neurons in the central ganglia (Faccioni-Heuser et al., 2004; Wyeth and Croll, 2011).

### 2.1 Identification of SoxB genes in the transcriptome of *Lymnaea stagnalis*

To identify SoxB-family genes, we examined the available partial transcriptome of *L. stagnalis*, sourced from a mix of postmetamorphic snails (st. 28–29) and adult nervous systems. Several Sox gene sequences were discerned based on the sequence of their HMG domains. Subsequent phylogenetic analysis classified them into the SoxB1, SoxB2, SoxD, SoxF, and SoxE subfamilies, aligning them with their respective orthologs from other Lophotrochozoans. The gene Pangolin, belonging to the Tcf family, was also identified and included as an outgroup. The identified SoxB genes distinctly clustered into the SoxB1 and SoxB2 groups (Figure 1g). These genes were designated as *LsSoxB1*, *LsSoxB2.* Both *L. stagnalis* SoxB-family sequences contain Sox-family KKDK and LPG conserved motifs. The *LsSoxB1* sequence has the TKT motif, while the *LsSoxB2* has the PKS motif within the HMG domain, identifying them as belonging to the SoxB1 and SoxB2 subfamilies, respectively (Supplementary Figure S1). The obtained sequences were used for the further work.

### 2.2 Specificity of Sox2-like immunoreactivity for *LsSoxB1*

**Figure 2:**
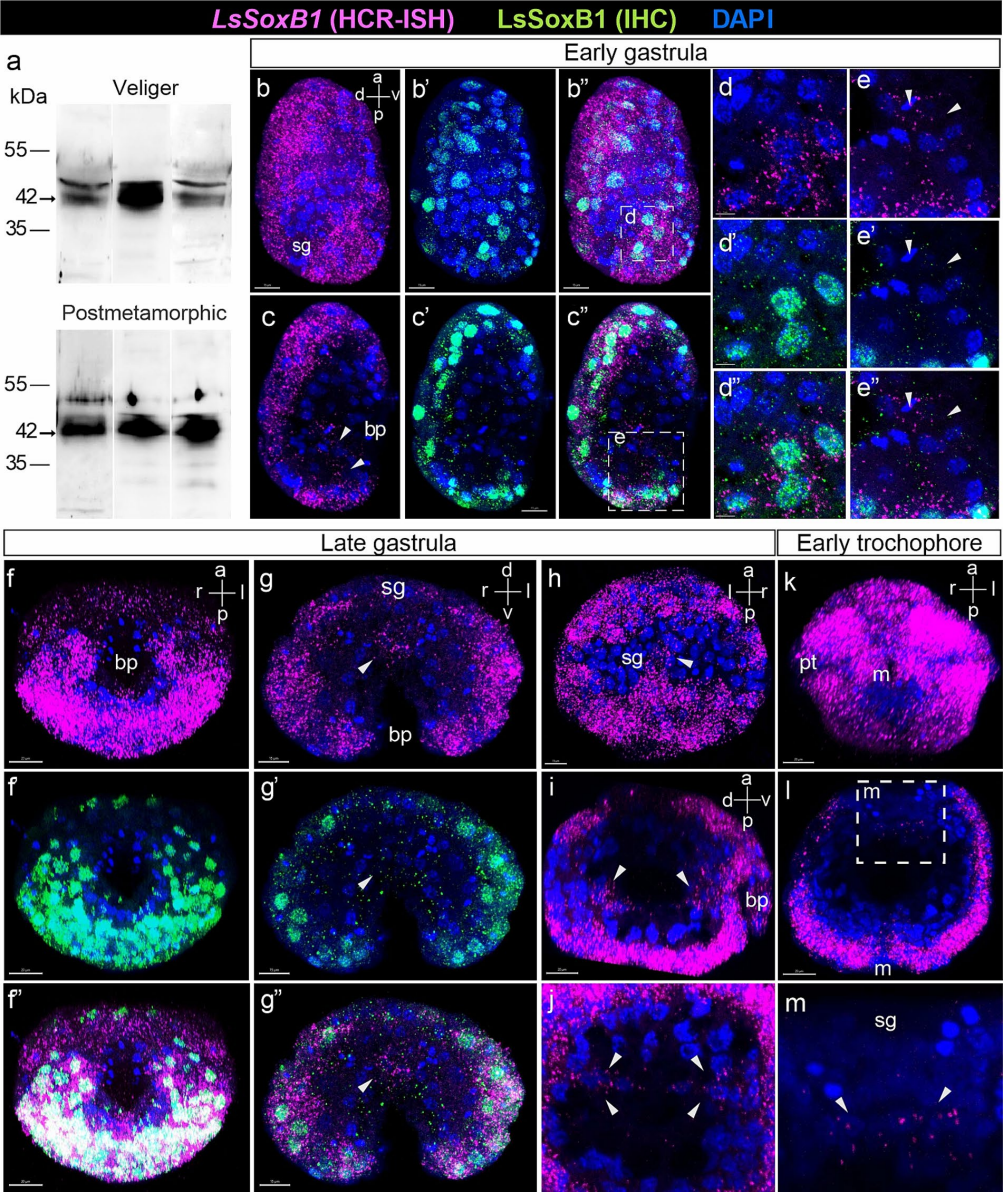
Expression of *LsSoxB1* in gastrula and early trochophore larvae. **(a)** The representative Western blot using rabbit antibodies against mouse Sox2 shows specific bands in lysates from *L. stagnalis* veliger larva and postmetamorphic snail. The consisted 42 KDa bands are present in three independent samples of each stage. **(b–b”)** Early gastrula side view, maximum projection and **(c–c”)** medial optical sections. Both *LsSoxB1* mRNA and protein are almost ubiquitously distributed in the most ectodermal cells. **(d–d”)** Enlarged images demonstrating the cytoplasmic localization of *LsSoxB1* mRNA and the nuclear localization of LsSoxB1 protein in ectoderm cells. **(e–e”)** Enlarged images of invaginating endodem. Arrowheads indicate the blastopore lip cells with exclusively *LsSoxB1* transcripts but not protein expression. **(f–f”)** Late gastrula ventral view, maximum projection, **(g–g”)** transverse optical section through the blastula, and **(h)** dorsal view, maximum projection. Both *LsSoxB1* mRNA and protein widely distributed in ectoderm excluding the shell gland anlage. Arrowheads indicate cells in the gut with *LsSoxB1* transcripts but not protein expression. **(i)** Sagittal optical section through the archenteron and **(j)** transversal optical sections through the posterior portion of the archenteron. Arrowheads indicate the cells expressing *LsSoxB1* mRNA in the wall of the forming gut. **(k)** Early trochophore ventral view, maximum projection. All ectodermal cells maintain a high signal intensity of *LsSoxB1*, with the exceptions of the zone ventrally to the mouth and visibly low signal in prototroch. **(l,m)** Transverse optical section and enlarged image of the forming gut. *LsSoxB1*-positive cells (arrowheads) in the part of the gut wall adjacent to the shell gland anlage. bp, blastopore; m, mouth opening; pt, prototroch; sg, shell gland anlage. Scale bars: b-c” – 15 µm, d,e – 5 µm, f-f”,i,k,l – 20 µm.

To comprehensively investigate *LsSoxB1* expression, encompassing potential translational regulation, we adopted a dual approach involving the analysis of both mRNA and protein expression. Fluorescent hybridization chain reaction *in situ* hybridization (HCR-ISH) for *LsSoxB1* was complemented with immunohistochemistry (IHC) utilizing antibodies targeting the epitope in the highly conserved part of the HMG domain in mouse Sox2. To validate the specificity of the antibodies employed, we performed Western blot analysis, confirming the presence of Sox2-immunoreactive (Sox2-IR) bands in veliger and postmetamorphic stages of *L. stagnalis*. Triple replicates revealed the consisted bands exhibited a molecular weight close to the predicted weight for LsSoxB1 protein based on its sequence (42 KDa; Figure 2a) in samples from veliger and postmetamorphic stages. Additionally, we detected bands at close regions of ~45 KDa and ~ 51 KDa (Figure 2a), possibly attributed to isoforms from alternative splicing, or posttranslational modifications of the protein.

We observed a high concordance between *LsSoxB1* mRNA expression and presence of LsSoxB1 protein in developing larvae at various stages (see Figures 1b–e”,f–f”,g–g”, 2h–h”,k–k”). This alignment underscored the reliability of Sox2 antibodies as a marker for LsSoxB1 protein in *L. stagnalis*, notwithstanding the presence of additional bands on the Western blot.

Interestingly, our exploration also identified minor regions lacking LsSoxB1 protein but exhibiting exclusive *LsSoxB1* mRNA expression. This intriguing finding prompted us to consider translational regulation, a phenomenon well-documented for SoxB1 orthologs in other animals (Angerer et al., 2005), as a potential explanation for this discrepancy. To further validate the specificity of Sox2-IR to LsSoxB1, we conducted co-staining with *LsSoxB2* visualized by HCR-ISH, revealing a distinct expression pattern that supported the selective nature of Sox2-IR in capturing LsSoxB1 dynamics in the developing gastropod nervous system for details, see later.

### 2.3 *LsSoxB1* expression prior to ganglia formation

During gastrulation, particularly in stages involving the invagination of the endoderm (early gastrula), *LsSoxB1* expression predominantly localized to ectodermal regions. All surface areas displaying *LsSoxB1* expression demonstrated co-occurrence with the presence of Sox2-IR (Figures 2b–c”). Remarkably, expression of mRNA coincides with the protein in ectoderm cells (Figures 2d–d”). In addition to ectodermal regions, *LsSoxB1* expression with visibly lower signal intensity was observed in the invaginating endodermal cells in the posterior blastopore lip, while the corresponding symmetrical anterior portion of the blastopore lip lacked this expression in early gastrula (Figures 2c–c”). It is noteworthy that while *LsSoxB1* mRNA was present in these specific cells in the archenteron (future foregut), the corresponding LsSoxB1 protein expression was not detected (Figures 2e–e”). The expression of *LsSoxB1* was not observed in the majority of endodermal cells in early gastrula, as well as in the compact region of ectodermal cells in the dorsal posterior zone, which refers to the further differentiation of the shell gland. In subsequent stages (late gastrula, early trochophore, high-intensity signal of both *LsSoxB1* mRNA) and protein was paralleled in ectodermal cells (Figures 2f–f”). Visibly lower signal intensity occurs in the anterior hemisphere and in the ventral zone around the mouth (Figures 2f–f”,k). Note that no *LsSoxB1* signal occurs in the shell gland formation zone at the dorsal embryo side (Figure 2h). Exclusively *LsSoxB1* expression but not the presence of LsSoxB1 protein was observed in cells of the gut wall (arrowheads in Figures 2i,j). *LsSoxB1*-positive cells locate in the wall of the forming gut adjacent to the shell gland formation prospective zone in early trochophore (arrowheads in Figures 2g–g”,l,m).

**Figure 3:**
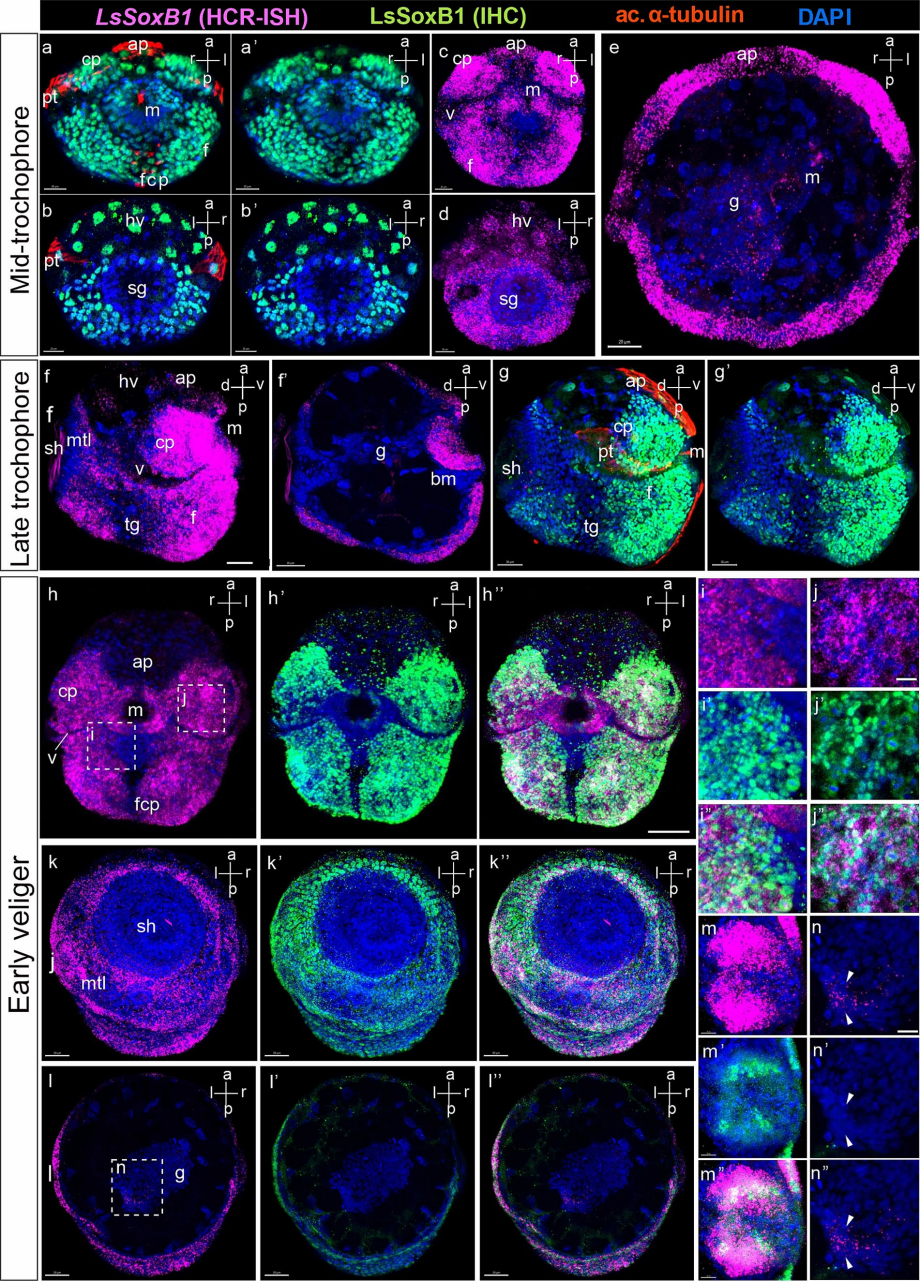
*LsSoxB1* expression in mid-trochophore, late trochophore and early veliger. Mid-trochophore ventral **(a,a’,c)** and dorsal views **(b,b’,d)** maximum projections. *LsSoxB1* transcript and protein widely distributed in ectoderm cells including cephalic plate and the entire surface of the foot rudiment. Prototroch, apical ciliated cells, head vesicles and cell surrounding the mouth demonstrate visibly lower signal intensity. **(e)** Transverse optical section. Note a ring of cells expressing *LsSoxB1* mRNA in the forming midgut. **(f,g,g’)** Late trochophore, side view, maximum projection. Sustained LsSoxB1 expression occurs throughout head and foot ectoderm, and visceral part ventrally to the shell, Prototroch and transverse foot groove cells lack *LsSoxB1* expression signal. **(f’)** Sagittal optical section. Note the expression of *LsSoxB1* in the oral cavity cells. **(h–h”)** Early veliger ventral views, **(k–k”)** dorsal views, maximum projection and **(l–l”)** transverse section. Note the sustained high expression level of *LsSoxB1* mRNA in cerebral plates, foot, and mantle, but not in the shell gland. **(i–i”)** Higher magnification of **(h)**. Note that some cells in the anterior foot area ventrally to the mouth show low *LsSoxB1* mRNA and high LsSoxB1 protein expression. **(j–j”)** High magnification of **(h)**. Note the co-expression of *LsSoxB1* mRNA and LsSoxB1 protein within the cells of the cerebral plate. **(m–m”)** Enlarged images of the oral cavity. Note that *LsSoxB1* transcript demonstrates broader expression than LsSoxB1 protein. **(n–n”)** Higher magnification of **(l)**, enlarged images of the midgut. Note the exclusive presence of *LsSoxB1* mRNA but not LsSoxB1 protein in some midgut cells. ap, apical plate; bm, buccal mass; cp, cerebral plate; f, foot; fcp, foot ciliary plate; g, gut; hv, head vesicle; m, mouth; mtl, mantle; pt, prototroch; sh, shell gland; tg, transverse foot groove. Scale bars: a-e – 20 µm, f-l” – 30 µm, i-n” – 10 µm.

At the mid-trochophore stage, high-intensity signal of both *LsSoxB1* mRNA and LsSoxB1 peptide expression were present in most ectodermal cells, including cephalic plate areas, the entire surface of the foot rudiment, and around the forming shell gland (but not in shell gland anlagen). A small area underneath the mouth opening demonstrated no signal expression (Figures 3a–d). A ring of small cells expressing *LsSoxB1* mRNA was located in the forming midgut in mid-trochophore (Figure 3e). At the late trochophore stage, visibly lower signal intensity occurred in the prototroch cells, apical ciliated cells, head vesicles and cells surrounding the forming mouth, and cells of transverse foot groove Both *LsSoxB1* mRNA and LsSoxB1 protein expression (Figures 3f,g,g’), while *LsSoxB1* mRNA high signal intensity appears in the mouth cavity walls (Figure 3f’). Notably, by the early veliger stage, signal practically disappears in the prototroch, apical cells and head vesicles and medial line cells ventral to the mouth opening (Figures 3h–h”). Both *LsSoxB1* mRNA and LsSoxB1 protein expression became prominent in the forming mantle around the shell gland (Figures 3k–k”). Expression of *LsSoxB1* remains in the midgut wall (Figures 3l–l”) in the portion of the gut adjacent to the shell gland (Figures 3n–n”).

Detailed examination revealed variations between *LsSoxB1* expression and the presence of LsSoxB1 protein in different larval tissues in early veliger. Notably, a *LsSoxB1* signal became lower in the foot region ventral to the mouth, coinciding with vibrant immunoreactivity of LsSoxB1 protein in the corresponding area (Figures 3i–i”). Moving to the cells of the cerebral plate, the cytoplasmic expression of *LsSoxB1* perfectly aligned with the nuclear localization of LsSoxB1 protein (Figures 3j–j”). In the oral cavity wall, the zone of *LsSoxB1* expression surpassed that of LsSoxB1, showcasing a gradient from surface to depth, extending from the mouth opening to the depth of the intestine (Figures 3m–m”). *LsSoxB1* mRNA expression persists in the midgut cells without a sign of LsSoxB1 protein (Figures 3n–n”).

### 2.4 *LsSoxB1* expression during ganglia formation and metamorphosis

**Figure 4:**
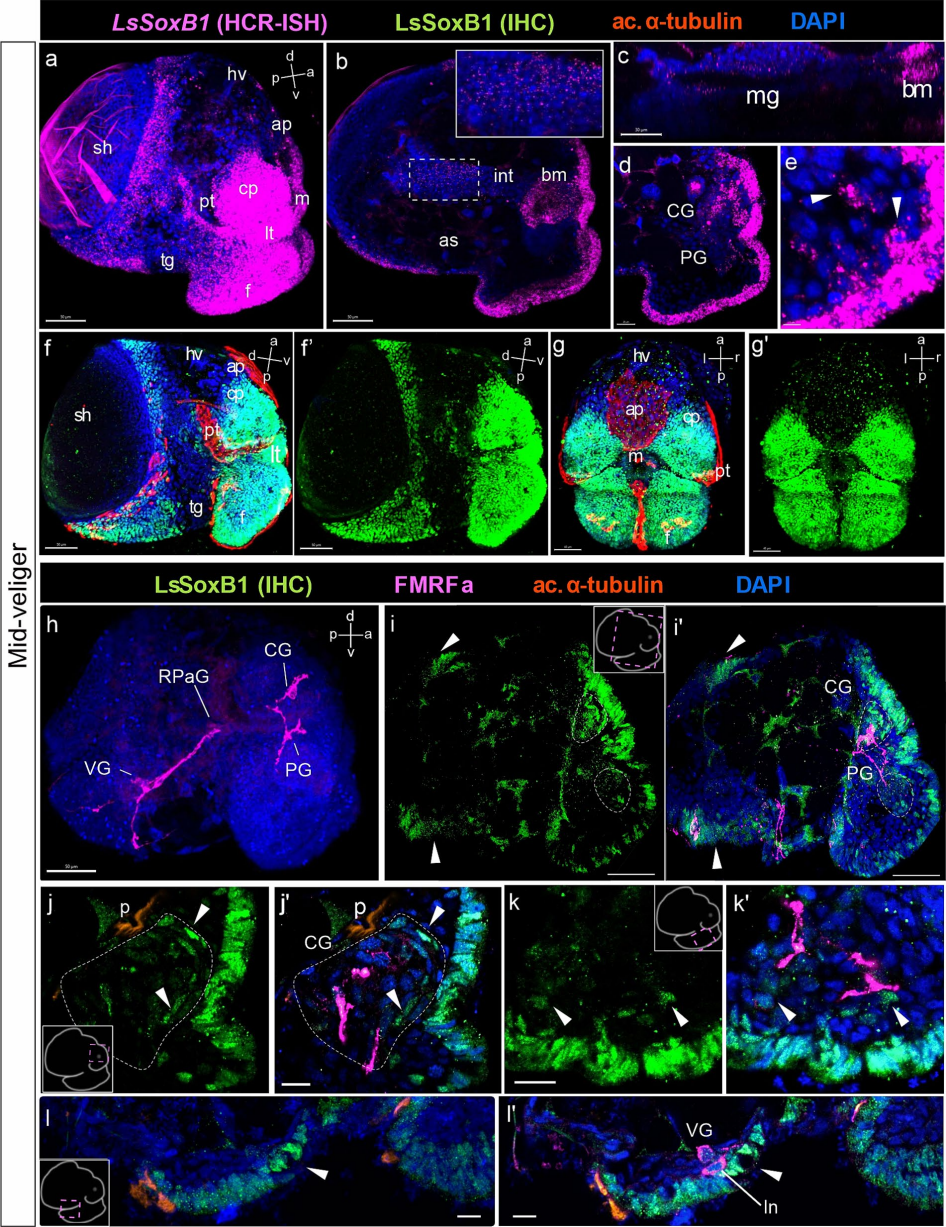
*LsSoxB1* expression in mid-veliger. Mid-veliger side view **(a,f,f’)** maximum projections, **(b,c)** sagittal and **(d,e)** parasagittal sections. **(g,g’)** Ventral views, maximum projection. Both *LsSoxB1* mRNA **(a)** and LsSoxB1 protein **(f–g’)** expressed in the head and foot ectoderm, mantle, and visceral ectoderm. Note the lack of signal in prototroch, head vesicle, apical plate, foot transverse groove and foot ciliary plate cells. *LsSoxB1* expression occurred in the mouth **(b)** and some of forming midgut cells **(c)**. *LsSoxB1*-positive cells appear in the forming cerebral ganglia **(d)**, arrowheads indicate the subepithelial cells in the foot **(e)**. **(h)** Mid-veliger, whole mount anti-FMRFamide IHC labeling (FMRFa). Processes of early FMRFa peripheral cells scaffold the neuropil of developing cerebral, pedal, right parietal, and visceral ganglia. **(i–l’)** Double labeling of LsSoxB1 and FMRFa immunoreactivity, cryosections. **(i,i’)** LsSoxB1-positive cells in head and foot, mantle and visceral epithelium (arrowheads). Note that in this stage LsSoxB1-positive cells present also in the forming cerebral ganglia. **(j,j’)** High magnification of the forming cerebral ganglia. Arrowheads indicate LsSoxB1-positive cells at the outer margin of the ganglion. **(k,k’)** High magnification of the foot region. Arrowheads indicate subepithelial LsSoxB1-positive cells beneath the LsSoxB1 epithelium in the foot. Some of the cells are adjacent to FMRFa fibers. **(l,l’)** High magnification of the visceral body part at the region of forming visceral ganglion. Note the wider distribution of *LsSoxB1* epithelial cells than area of presumptive ganglion formation. ap, apical plate; af, autofluorescence; as, albumen sac; bm, buccal mass; CG, cerebral ganglion; cp, cephalic plate; f, foot; hv, head vesicle; int., intestine; ln, left peripheral neuron; lt, labial tentacle; m, mouth; mg, midgut; p, protonephridia; pt, prototroch; PG, pedal ganglion; RPaG, right parietal ganglion; sh, shell; tg, transverse foot groove; VG, visceral ganglion. Scale bars: a,b,d,f-i’ – 50 µm, c – 30 µm, e,j-k’ – 10 µm, l,l’ – 20 µm.

Throughout the mid-veliger stage, both *LsSoxB1* mRNA and protein expression persisted across the extensive ectodermal area of the head, including the tentacles, as well as the dorsal, lateral, and ventral surfaces of the foot ectoderm. Additionally, LsSoxB1 expression was observed in the mantle and visceral ectoderm areas, except the prototroch, foot grove and head vesicle cells (Figures 4a,f–g’). Notably, *LsSoxB1* was present in the oral cavity and the wall cells of the developing midgut in mid-veliger (Figures 4b,b’,c), with a few cells located in the region of the forming cerebral ganglia (Figure 4b) and cells in the subepithelial layer in the foot (Figure 4e).

To further investigate *LsSoxB1* expression and its association with differentiated neural elements, we conducted combined anti-LsSoxB1 protein immunostaining (LsSoxB1 IHC) and FMRFamide. At the mid-veliger stage, FMRFamide-like immunoreactivity (FMRFa) highlighted early peripheral cells (caudal, left, and right peripheral neurons) and neuropil in forming ganglia (Figure 4h). LsSoxB1 was observed in the cells of visceral epithelium adjacent to the left and right early FMRFa neurons (arrowheads in Figures 4i,i’), as well as in the epithelial layer cells above the forming cerebral ganglion (Figures 4i,i’) and adjacent to the forming visceral ganglion (Figures 4l,l’). Notably, several LsSoxB1-containing cells were located at the outer margin of the forming cerebral and pedal ganglia beneath the LsSoxB1-positive epithelium of the cephalic plate and foot, respectively (Figures 4j,j’,k’,k’). In addition, some subepithelial cells located between the forming pedal ganglia and the LsSoxB1-positive foot epithelium (Figures 4k,k’).

**Figure 5:**
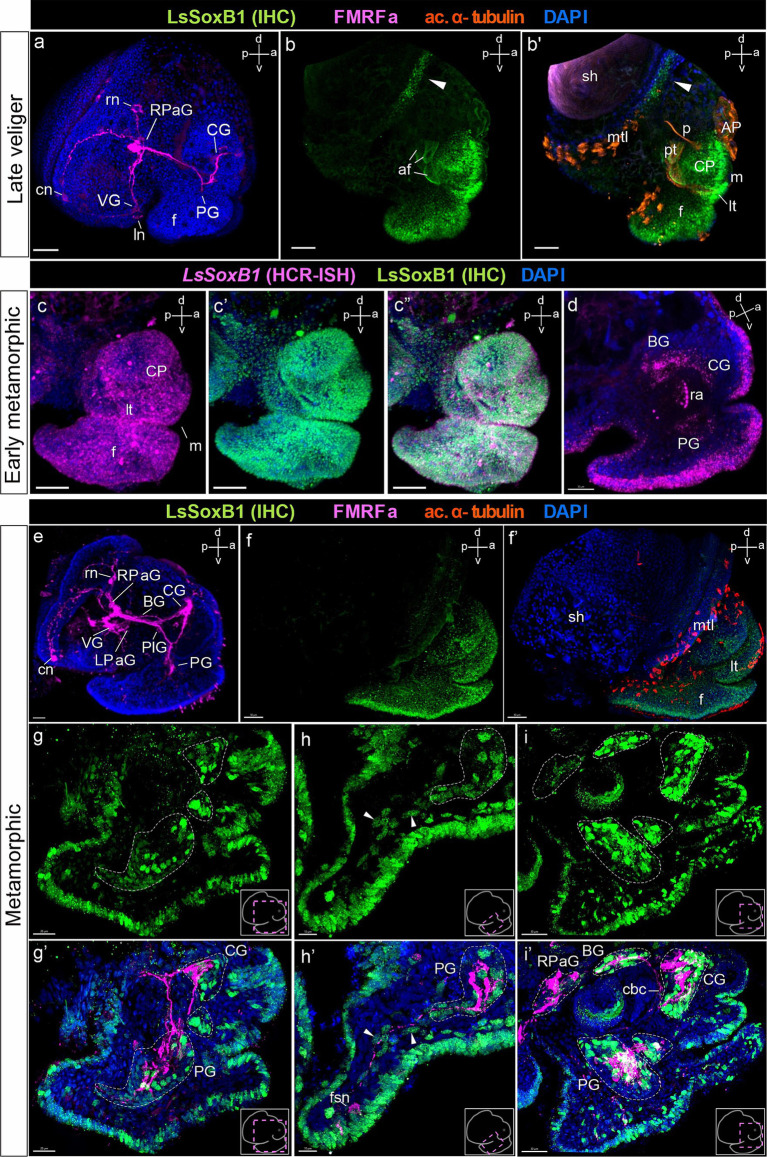
*LsSoxB1* expression in late veliger, and metamorphic larvae. **(a)** Side view of late veliger, whole mount anti-FMRFa IHC labeling. FMRFa-positive solitary cells located in forming parietal and visceral ganglia. FMRFa processes mark neuropil of developing cerebral and pedal ganglia. **(b,b’)** Late veliger side view, maximum projection. Note persistent extensive epithelial expression of LsSoxB1 in the head and foot epithelium and appearance of LsSoxB1-positive area at the mantle margin (arrowheads). **(c–c”)** Early metamorphic larva side view, maximum projection. Both *LsSoxB1* transcripts and LsSoxB1 protein are widely expressed in the epithelia of the head and foot. **(d)** Early metamorphic larva, sagittal optical section. *LsSoxB1* expression occur in solitary cells of forming cerebral, pedal, and buccal ganglia. **(e)** Metamorphic larva, whole-mount a-FMRFa IHC labeling. FMRFa processes mark the central ring ganglia neuropile and interconnecting connectives and commissures. **(f,f’)** Metamorphic larva side view, maximum projection. Extensive surface distribution of LsSoxB1 remains throughout the head, tentacles, and foot surface epithelia. **(g–i’)** Double labeling of LsSoxB1 and FMRFa immunoreactivity, cryosections. **(g,g’)** Parasagittal section through the cerebral and pedal ganglia. Note LsSoxB1-positive cells along the anterior margin of the cerebral and pedal ganglia, together with LsSoxB1-positive cells in epithelium of foot, lip, and tentacles. **(h,h’)** Parasagittal section through the foot. Arrowheads indicate LsSoxB1-positive subepithelial cells right underneath the epithelium and deep in the foot. Some of the cells associated with FMRFa-positive fibers. Note that FMRFa-positive sensory neuron in the foot is lack of LsSoxB1 expression. **(i,i’)** Parasagittal oblique section through the central ring ganglia. LsSoxB1-positive reaction remains in epithelial and subepithelial cells simultaneously with numerous LsSoxB1-positive cells in ganglia. Note that all ganglia except the right parietal contain LsSoxB1-positive cells. AP, apical plate; af, autofluorescence; BG, buccal ganglion; cbс, cerebro-buccal commissure; CG, cerebral ganglion; cn, central early peripheral neuron; CP, cephalic plate; f, foot; fns, foot sensory neuron; ln, left early peripheral neuron; LPaG, left parietal ganglion; lt, labial tentacle; m, mouth; mtl, mantle; p, protonephridium; PlG, pleural ganglion; pt, prototroch; PG, pedal ganglion; RPaG, right parietal ganglion; rn, right early peripheral neuron; ra, radular sac; sh, shell; VG, visceral ganglion. Scale bars: a-b’,e – 50 µm, c-d,g,g’ – 30 µm, f – 70 µm, h,h’ – 10 µm, i,i’ – 20 µm.

At the late veliger stage, FMRFa highlighted the scaffold of the forming nervous system and solitary cells differentiated in parietal and visceral ganglia (Figure 5a). High-intensity LsSoxB1 signal persisted in the outermost epithelium of the head, labial tentacles, and foot (Figures 5b,b’) and the margin of the mantle (indicated by an arrowhead in Figures 5b,b’). A similar pattern of LsSoxB1 protein and *LsSoxB1* mRNA expression was maintained at the early metamorphic stage epithelia (Figures 5c–c”) and in cells of the central ganglia (Figure 5d).

At the early metamorphic stage, the presence of all ganglia was distinctly marked by FMRFa-positive cells and the presence of neuropil (Figure 5e). LsSoxB1-positive cells still constituted a continuous layer in the head, tentacles, and foot epithelium (Figures 5f,f’), and present in cells located along the anterior margin of the cerebral and pedal ganglia (Figures 5g,g’). A subset of subepithelial LsSoxB1 cells in the foot was associated with FMRFa-positive processes originating from the pedal ganglia (arrowheads in Figures 5h,h’). Note that FMRFa-positive foot sensory neurons are LsSoxB1-negative (Figure 5h’). LsSoxB1-positive cell nuclei are presented in the cerebral, pedal, and buccal ganglia, but not in the parietal ganglion (Figures 5i,i’).

**Figure 6:**
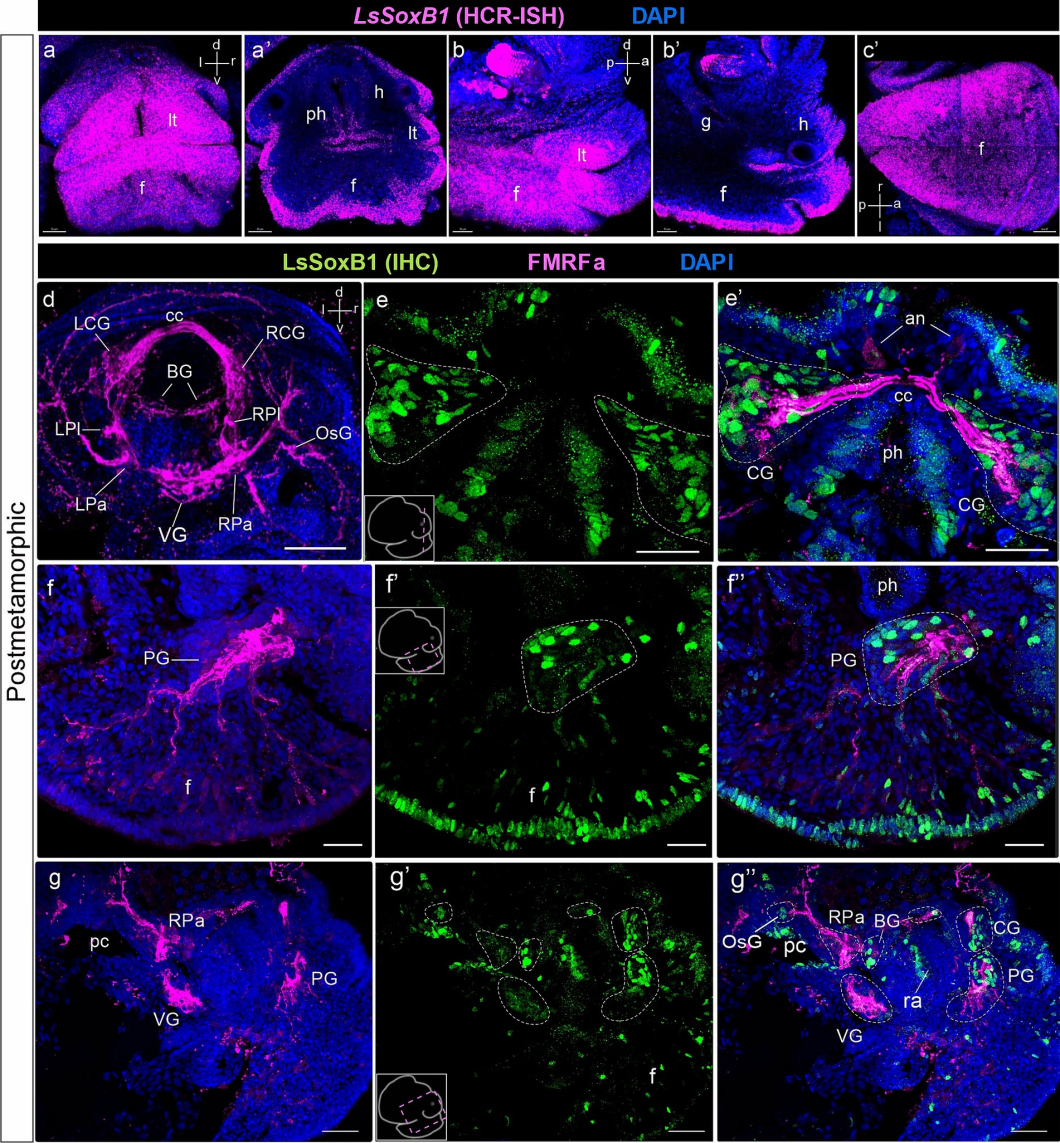
*LsSoxB1* expression after metamorphosis. Postmetamorphic adult-like snail frontal **(a,a’)**, side **(b,b’)** and ventral **(c)** views. **(a–c)** maximum projections, **(a’)** frontal optical section, **(b’)** parasagittal optical section. *LsSoxB1* expression is maintained in the head and foot epithelia, labial tentacles, dorsal and ventral foot surfaces. **(d)** Central ring ganglia of postmetamorphic adult-like snail, dorsal view, whole mount anti-FMRFa IHC labeling. FMRFa expressing cells and processes mark all ganglia of the nervous system, interconnecting commissures and connectives and solitary cells in ganglia. **(e–g’)** Double IHC labeling of LsSoxB1 and FMRFa, performed on cryosections. **(e,e’)** Section through cerebral ganglia. Numerous LsSoxB1-positive cells are observed in the ganglia cortical layer, along with LsSoxB1-positive cells in the head epithelium. **(f–f”)** Parasagittal section at the level of the pedal ganglion. LsSoxB1-positive nuclei are noted in the ganglia cortical layer, concurrently with sustained LsSoxB1 reaction in the cells of the foot epithelium and subepithelial layer. Some of the subepithelial LsSoxB1-positive cells are located along the FMRFa-positive fibers. **(g–g”)** Parasagittal section through the central ring ganglia. LsSoxB1-positive cells are present in the cerebral, pedal, buccal, and osphradial ganglia, but absent in the visceral and right parietal ganglia. an, apical neuron; BG, buccal ganglion; cс, cerebral commissure; CG, cerebral ganglion; h, head region; f, foot; g, gut; LCG, left cerebral ganglion; LPa, left parietal ganglion; LPl, left pleural ganglion; lt, labial tentacle; OsG, osphradial ganglion; pc, pulmonary cavity; ph, pharynx; PG, pedal ganglion; PlG, right pleural ganglion; RCG, right cerebral ganglion; RPa, right parietal ganglion; RPl, right pleural ganglion; ra, radular sac; VG, visceral ganglion. Scale bars: a-c,f-f’ – 20 µm, d – 50 µm, e,e’ – 30 µm, g-g” – 40 µm.

At the postmetamorphic adult-like stages, *LsSoxB1* maintained extended expression across areas of the head and foot epithelium (Figures 6a,a’,b,b’). The area with *LsSoxB1* visibly higher intensity signal located as a rim along the edge of the foot sole epithelium (Figure 6c). At this stage, the central ring ganglia were fully formed and concentrated around the esophagus (Figure 6d). Numerous LsSoxB1-positive neurons are observed in the cortical layer of cerebral (Figures 6e,e’) and pedal ganglia (Figures 6f–f”). Solitary cells with high-intensity signal expression remained in the layer beneath the epithelium. To the contrary, solitary subepithelial LsSoxB1-positive cells associated with pedal FMRFa-positive nerve bundles demonstrate visibly lower signal intensity (Figures 6f–f”). In the nervous system, LsSoxB1-positive cell bodies were present in the cerebral, pedal, and buccal ganglia, and peripheral osphradial ganglia. Notably, only some LsSoxB1 processes but not cell nuclei are present in the visceral and right parietal ganglia (Figures 6g–g”).

### 2.5 Proliferative activity of *LsSoxB1* expressing cells

Given the well-established role of SoxB1 in sustaining the proliferative activity of proneural cells, it is noteworthy that SoxB1 serves as a distinctive marker of pre-mitotic neuroblasts (Karnavas et al., 2013; Deryckere et al., 2021). This marker aids in identifying both the primarily proliferative zones in the neurogenic epithelium and secondary proliferative zones in the CNS. To assess the proliferative capacity of LsSoxB1-expressing cells situated in both the epithelial and subepithelial layers, we conducted investigations into their ability to incorporate 5-ethynyl-2-deoxyuridine (EdU) and undergo proliferation, as evidenced by immunostaining with antibodies against phosphorylated histone H3 (pH3). Our investigation spanned the late trochophore, mid-veliger, and metamorphic stages.

**Figure 7:**
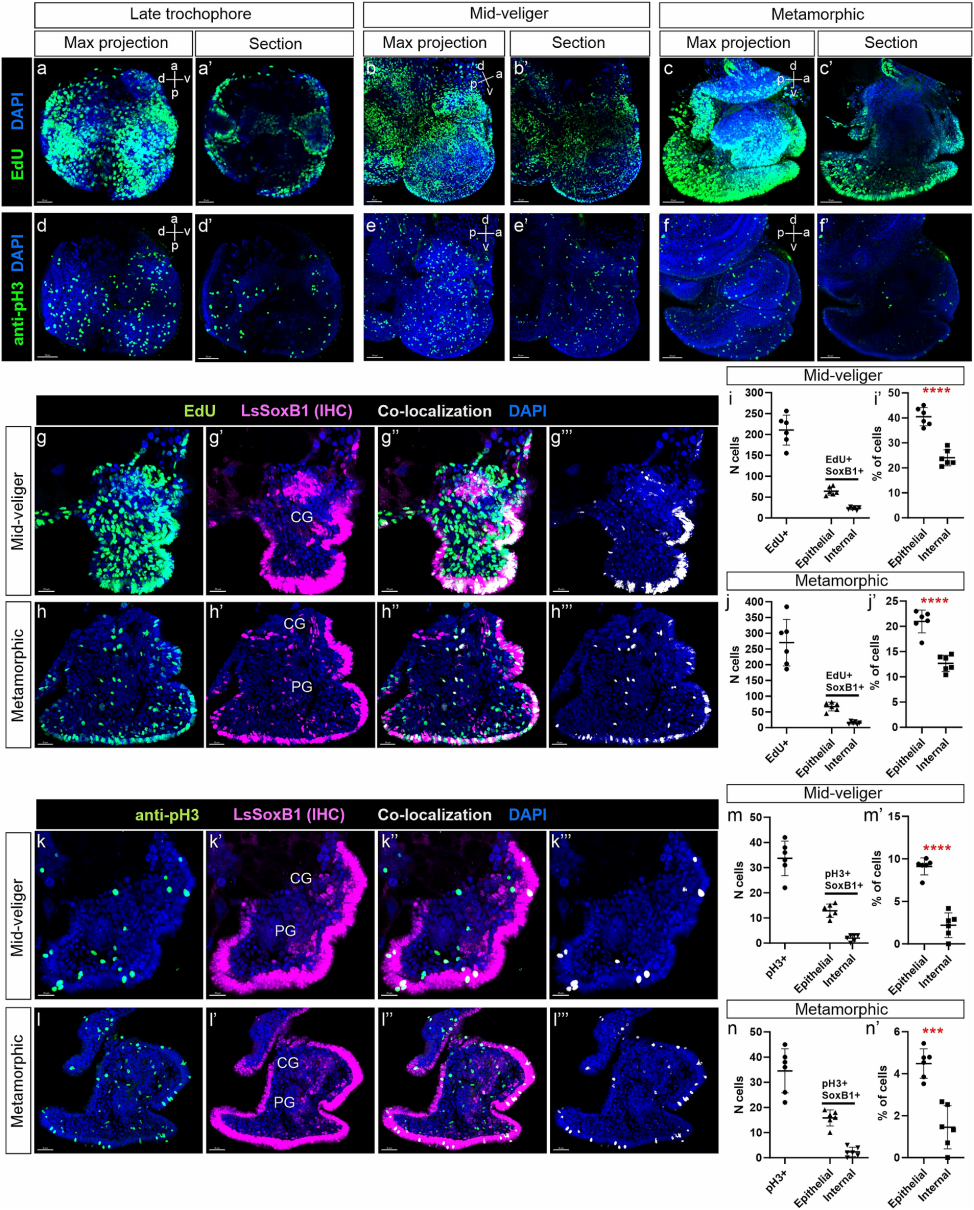
Analysis of *LsSoxB1* cells proliferative capacity in veliger and metamorphic larvae. **(a–c’)** Visualization of EdU incorporation after a 5-min pulse, and **(d–f’)** expression of phosphorylated histone H3 (pH3) in late trochophore, mid-veliger, and metamorphic larvae. Images **(a–f)** show maximum projections, while **(a’–f’)** depict sagittal sections. It is noteworthy that EdU incorporation is prominently observed, and pH3-positive cells are evenly distributed. **(g–h”’)** Double labeling of LsSoxB1 (LsSoxB1 IHC) and EdU assay, conducted on cryosections of mid-veliger and metamorphic larvae. Co-localization of LsSoxB1 and EdU is depicted in **(g”’,h”’)**. **(i,i’,j,j’)** Proportion of LsSoxB1/EdU-positive cells in epithelial and internal regions of mid-veliger and metamorphic larvae. **(k’–k”’,l–l”’)** Double IHC labeling of LsSoxB1 and pH3, performed on cryosections of mid-veliger and metamorphic larvae. Co-localization of LsSoxB1 and pH3 is presented in **(k”’,l”’)**. **(m,m’,n,n’)** Proportion of LsSoxB1/pH3-positive cells in epithelial and internal regions of mid-veliger and metamorphic larvae. It is observed that dividing LsSoxB1-positive cells are preferentially located in the epithelium, and their number decreases with age. *T*-test results indicate statistical significance with *** denoting *p* = 0.001 and **** denoting *p* < 0.001. CG, cerebral ganglion; PG, pedal ganglion. Scale bars: a,a’,d,d’,g-g”’ – 20 μm, b,b’,e,k-k”’ – 30 μm, c,c’,f,f’,h-h”’,l-l”’ – 50 μm.

Following a 5-min incubation with EdU, numerous EdU-positive cells were distributed throughout the embryonic body at all examined stages. Notably, at the late trochophore and metamorphic stages, the labeled cells were primarily located within the epithelia (Figures 7a,a’,c,c’). However, in the mid-veliger stage, numerous EdU-positive cells were also found in the internal subepithelial layer (Figures 7b,b’). Importantly, the EdU-incorporating cells were evenly distributed without the formation of distinct zones or clusters of EdU-positive cells in the embryonic body. This uniform pattern persisted across all developmental stages examined (Figures 7a–c’), with some EdU-positive cells also found within the areas of forming ganglia.

Immunohistochemical visualization of dividing cells with antibodies against pH3 revealed a significantly lower number of cells compared to those incorporating EdU (Figures 7d–f’). Similar to EdU labeling, pH3-positive cells were evenly distributed across the epithelium and subepithelial layers in mid-veliger and metamorphic animals. Importantly, no specific zones with concentrated pH3-positive cells were observed at any of the examined stages (Figures 7d–f’).

Further analysis, involving double labeling with antibodies against Sox2 and EdU incorporation assay at mid-veliger and metamorphic stages (Figures 7g’–g’”,h–h’”), revealed that 40.5% in veliger and 21% in metamorphic of epithelial LsSoxB1-positive cells were EdU-positive, while only 24% in veliger and 12.7% in metamorphic of the internal LsSoxB1-expressing cells were EdU-positive cells (Figures 7i,i’,j,j’). In both analyzed stages, some internal LsSoxB1/EdU-positive cells corresponded to the areas of forming ganglia.

Double labeling with antibodies against Sox2 and pH3 revealed the presence of rare LsSoxB1/pH3-positive cells in both the epithelium and the internal layer (Figures 7k–k’”,l–l’”). Few cells co-expressing LsSoxB1 and pH3 were found in the cortical layer of cerebral and pedal ganglia. LsSoxB1/pH3-positive cells were more prevalent in the epithelial layer (9.1 and 4.5% at veliger and metamorphic stages, respectively) than in the internal layer (2.2% in veliger and 1.5% in metamorphic) (Figures 7m,m’,n,n’). Thus, both methods demonstrate the presence of approximately twice as many proliferative LsSoxB1-positive cells in the epithelial layer than in the internal layer, indicating a decrease in the number of dividing LsSoxB1-positive cells during development.

### 2.6 *LsSoxB2* expression and its co-expression with *LsSoxB1*

**Figure 8:**
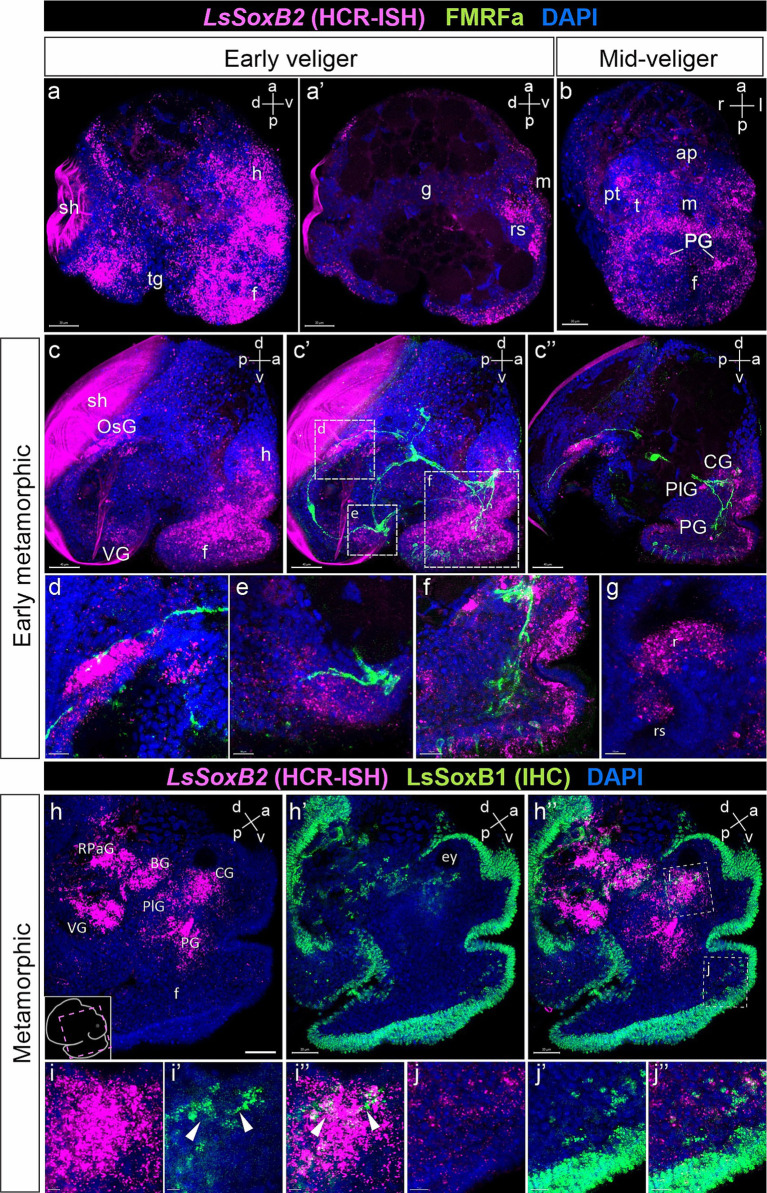
*LsSoxB2* expression in relation to FMRFamide-containing nerve elements and *LsSoxB1*. **(a)** Early veliger side view, maximum projection and **(a’)** sagittal section. Note the extensive expression of *LsSoxB2* in the majority of ectodermal cells excluding the shell gland and transverse foot groove cells. Also, note the *LsSoxB2* expression in the differentiating epithelium of the radular sac. **(b)** Mid-veliger ventral view, maximum projection. *LsSoxB2* is expressed in forming pedal ganglia, subepithelial layer cells of the head, and in the epithelium along the outer edge of the foot. **(c–f)** Double labeling of *LsSoxB2* mRNA *in situ* hybridization (HCR-ISH) and FMRFamide immunostaining (FMRFa). **(c,c’)** Early metamorphic larvae side view, maximum projection and **(c”)** parasagittal section. *LsSoxB2* expression present only in limited zones of epithelium. Note the presence of *LsSoxB2*-positive cells in ganglia rudiments marked by FMRFa processes and the foot subepithelial layer. **(d)** High magnification of the osphradial ganglion area. *LsSoxB2*-positive cells condense adjacent to the FMRFa fibers in the area of forming osphradial ganglion. **(e)** High magnification of the visceral ganglion area. *LsSoxB2*-positive cells around the FMRFa-positive fibers in neuropil. **(f)** High magnification of the cerebral and pedal ganglia zones. *LsSoxB2*-positive cells located in ganglia marked by FMRFa-positive fibers. **(g)** High magnification of the forming radula region, sagittal optical section. *LsSoxB2*-positive cells located at the ventral region of the radular sac. **(h–h”)** Metamorphic larvae side view, parasagittal section through the ganglia, *LsSoxB1* and *LsSoxB2* expression. **(h)** *LsSoxB2*-positive cells concentrates in the zones of forming central ring ganglia. **(h’)** *LsSoxB1* positive signal preferentially present in the epithelium. **(h”)** Visible difference in ganglionic expression of *LsSoxB2* and preferentially epithelial expression of *LsSoxB1.* **(i–i”)** High magnification of cerebral ganglia region. Arrowheads indicate solitary *LsSoxB1/LsSoxB2*-positive cells. **(j–j”)** High magnification of foot region. Note the absence of *LsSoxB2* positive cells in the epithelium while both *LsSoxB1* and *LsSoxB2* positive cells present in the subepithelial layer. ap, apical plate; BG, buccal ganglion; CG, cerebral ganglion; ey, eye; f, foot; g, gut; h, head; m, mouth; OsG, osphradial ganglion; pt, prototroch; PG, pedal ganglion; PlG, pleural ganglion; rs, radular sac; RPaG, right parietal ganglion; sh, shell; tg, transverse foot groove; t, tentacle; VG, visceral ganglion. Scale bars: a,a’,h-h” – 40 µm, b-c” – 50 µm, d,e,h – 10 µm, f, i-j” – 20 µm.

SoxB2, a member of the Sox gene family is known for its conserved role in establishing neural fate acting in coordination with proneural SoxB1. *LsSoxB2* expression begins by the early veliger stage, and the pattern of *LsSoxB2* closely resembles that of the *LsSoxB1*, extensively distributed in the epithelium, covering broad areas of the head, foot, visceral mass, and mantle (Figure 8a). Beyond the epithelium, *LsSoxB2* is expressed in the ventral part of the oral cavity and the dorsal wall of the radular sac rudiment (Figure 8a’). Subsequently, the signal intensity became lower in ectodermal cells but demonstrate higher signal intensity in the subepithelial cells of the head and foot, as well as in the forming pedal ganglia (Figure 8b). It is noteworthy that the zone of *LsSoxB2*-positive cells in the foot epithelium loses ubiquitous distribution and forms a rim along the outer edge (Figure 8b).

By the early metamorphic stage, *LsSoxB2* expression is limited to specific ectodermal areas, including the right mantle margin (the region where the osphradial ganglion forms), the ventral part of the visceral mass (adjacent to the forming visceral ganglion), the lateral edges of the foot, and the anterior portion of the tentacles (Figures 8c,c’). Internalized *LsSoxB2*-positive cells are found in areas corresponding to the forming pedal, cerebral, and osphradial ganglia, as well as in the subepithelial layer of cells in the foot (Figure 8c”). *LsSoxB2*-positive cells are adjacent to FMRFa-positive fibers, which mark the osphradial ganglion in mantle region (Figure 8d), visceral ganglion (Figure 8e), as well as the pedal and cerebral ganglia (Figure 8f). Additionally, a mass of subepithelial *LsSoxB2*-positive cells are located in the foot, and some of these cells express FMRFa (Figure 8f). In the oral cavity, *LsSoxB2* is expressed in the ventral region of the radular sac (Figure 8g).

At metamorphic stage, *LsSoxB2* signal almost disappears in the ectoderm but demonstrate high intensity signal within ganglia and in some subepithelial layer cells (Figure 8h). This ganglionic localization of *LsSoxB2* strongly contrasts with the predominantly epithelial localization of *LsSoxB1* (Figures 8h–h”). Solitary cells expressing both *LsSoxB1* and *LsSoxB2* can be found in the cerebral ganglia (Figures 8i–i”) and in subepithelial layer cells in the foot (Figures 8j–j”).

## 3 Discussion

**Figure 9:**
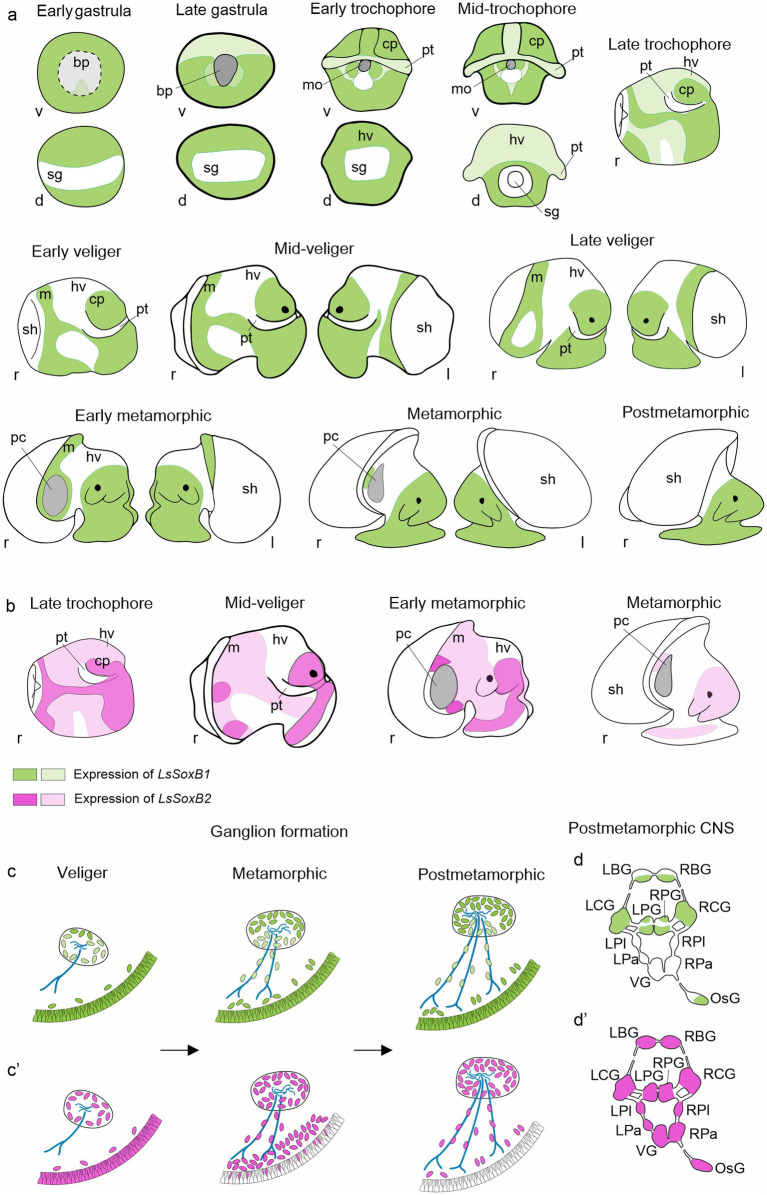
Summary of SoxB genes expression in the development of gastropod mollusk *Lymnaea stagnalis*. **(a)** Schematic representation of ectodermal expression of *LsSoxB1* from early gastrula to postmetamorphic snail. **(b)** Schematic representation of ectodermal expression of *LsSoxB2* at representative developmental stages. Notably, epithelial *LsSoxB2* expression commences later, by the late trochophore stage, compared to *LsSoxB1* expression, which begins in the early gastrula stage. Although their expression patterns are similar until the early veliger stage, *LsSoxB1* maintains epithelial expression throughout the mid-veliger, metamorphic, and even postmetamorphic stages. Meanwhile, *LsSoxB2* expression is limited to zones above the forming ganglia during mid-veliger and metamorphic stages and diminishes in the epithelial layer in the postmetamorphic stage. **(c,c’)** Scheme illustrating the distribution of *LsSoxB1* **(c)** and *LsSoxB2*-positive **(c’)** cells during gangliogenesis using the example of the pedal ganglion. In the veliger stage, both *LsSoxB1*- and *LsSoxB2*-positive cells are present preferentially in the epithelium and in the forming ganglia. However, by the metamorphic and postmetamorphic stages, *LsSoxB1* expression is confined to the epithelial layer and ganglia cells, and is also observed along the nerves. In contrast, *LsSoxB2* expression vanishes from the epithelium and is concentrated in the subepithelial layer at the metamorphic stage. At the postmetamorphic stage, *LsSoxB2*-positive cells disappear from the epithelium and are located along nerves and in ganglia only. **(d,d’)** Outlined distribution of *LsSoxB1* **(d)** and *LsSoxB2* **(d’)** expression in the ganglia of central nervous system in postmetamorphic snail. Among the central ring ganglia of the adult-like nervous system, the paired pleural and parietal ganglia, and visceral ganglion lack *LsSoxB1* expression. Green indicates *LsSoxB1* expression, while magenta represents *LsSoxB2* expression. Dark green and dark magenta signify visibly higher expression, while light green and light magenta indicate visibly lower expression. Blue indicates nerves. “d” signifies the view from the dorsal side, “l” from the left side, “r” from the right side, and “v” from the ventral side. bp, blastopore; cp, cephalic plate; f, foot; hv, head vesicle; LBG, left buccal ganglion; LCG, left cerebral ganglion; LPa, left parietal ganglion; LPG, left pedal ganglion; LPl, left pleural ganglion; mo, mouth opening; m, mantle; OsG, osphradial ganglion; pt, prototroch; RBG, right buccal ganglion; RCG, right cerebral ganglion; RPa, right parietal ganglion; RPG, right pedal ganglion; RPl, right pleural ganglion; sg, shell gland; sh, shell; VG, visceral ganglion.

Our study represents the first comprehensive report on the expression of SoxB family genes in the course of gastropod mollusk *L. stagnalis* development. Our data encompass the entire larval development, from gastrulation to the adult-like snail, including the trochophore, veliger and metamorphic stages. It reveals an extended pattern of LsSoxB1 expression in the ectoderm maintained both spatially and temporally (Figure 9a). Expression of LsSoxB1 is supported at both the transcriptional and translational levels, even in postmetamorphic stages that possess fully formed central ganglia and peripheral neuronal sensory elements. Significantly, a substantial portion of SoxB1-positive ectoderm is not associated with larval neurogenic areas. In addition, certain cells in the foregut and midgut from the gastrula stage onwards express *LsSoxB1* solely at the mRNA level and do not contain the corresponding protein. During ganglia formation and axonogenesis, only subsets of specific cells within ganglia and the solitary cells in foot express LsSoxB1 (Figures 9c,d). Meanwhile, the extended expression of *LsSoxB2* in the ectoderm is rapidly terminated at veliger stage (Figure 9b) and later remains in differentiating neurons in ganglia anlages and in subepithelial cells of the foot (Figures 9c’,d’). These results indicate that the expression pattern of the SoxB transcription factor family in the *L. stagnalis* differs significantly from other investigated invertebrates.

### 3.1 Similarities and differences with other animals

In most bilaterian animals, ectodermal SoxB1 expression during organogenesis is linked to the patterning of the neurectoderm and subsequent neurogenesis (Hartenstein and Stollewerk, 2015). In non-bilaterian cnidarians, as well as bilaterian platyhelminths lacking solid neuroectodermal zones, SoxB1-positive cells are scattered throughout the ectoderm without forming a continuous layer (Magie et al., 2005; Semmler et al., 2010; Richards and Rentzsch, 2014; Monjo and Romero, 2015). In annelids and arthropods, SoxB1 expression in the early stages of neurogenesis is limited to the neuroectodermal placodes, which are distinctly separated from the surrounding ectoderm and lie directly above the emerging structures of the central nervous system (Buescher et al., 2002; Simionato et al., 2008; Wilson and Dearden, 2008; Kerner et al., 2009; Sur et al., 2020). In all cases described, the neuroectoderm-committed areas are characterized by the presence of actively dividing cells.

However, pattern of SoxB1 expression in ectoderm of gastropod *L. stagnalis* contrasted with that in annelids and arthropods, in which it concentrated in clearly delineated and limited areas. In *L. stagnalis*, *LsSoxB1* expression occupies most of the head, foot, and visceral complex ectoderm. Our observations in *L. stagnalis* indicate that *LsSoxB1* expression definitely covers presumptive neurogenic ectoderm zones starting from the late trochophore and early veliger stages. Increased levels of *LsSoxB1* expression occur in the cerebral plates (cerebral ganglia anlagen), the ventral surface of the forming foot (pedal ganglia anlagen), and body wall near the areas of visceral, parietal, and osphradial ganglia anlagen. Moreover, *LsSoxB1* remains in epithelia by late postmetamorphic stages when the most larval ganglia and the most sensory periphery is already formed, and thus is not coincide with known events of nervous system differentiation. Such extensive widespread and prolonged expression of *LsSoxB1* in *L. stagnalis* suggests that in mollusks, SoxB1 may have an additional function beyond its role in neuroectoderm commitment. Such roles of SoxB1 in some non-neurogenic cells are known both for invertebrates and vertebrates. For example, in *Drosophila*, SoxNeuro (ortholog of SoxB1) takes part in shaping the denticles in the embryonic epidermis (Rizzo and Bejsovec, 2017). In *Xenopus*, Sox3 (one of SoxB1 orthologs in vertebrates) is an important factor in the differentiation of non-neural ectodermal cells in the neural plate border (Schock et al., 2023). However, unlike in the gastropod, these events take place in limited sites of the embryonic ectoderm only, and do not expand to the later developmental stages.

*LsSoxB1* is also present in zones corresponding to differentiating neurons at stages from late veliger to metamorphosis. However, *LsSoxB1*-positive cells never constitute the majority in the ganglia at any stage. Noteworthy, parietal and visceral ganglia contain *LsSoxB1*-positive processes only but not the *LsSoxB1* neural cell bodies. This observation aligns with studies in cephalopods, where SoxB1 transcripts were highly expressed in cerebral and pedal cord derivatives but absent in the palliovisceral cord (Focareta and Cole, 2016; Deryckere et al., 2021). Altogether, these results indicate that the late role of SoxB1 in the differentiation of neurons in mollusks is restricted to certain neuronal subtypes and is not attributed to all neurons.

In addition to the role in the development of central nervous system, SoxB1 genes are known to be involved in the differentiation of peripheral sensory cells (Ross et al., 2018). Earlier, the broad expression of SoxB1 and SoxB2 in various epithelial zones of the cuttlefish *Sepia officinalis* has been attributed to the extensive development of peripheral sensory structures in cephalopods (Buresi et al., 2014; Focareta and Cole, 2016). We observed extensive *LsSoxB1* expression in the epithelial areas of gastropod *L. stagnalis* throughout larval development, including the post-metamorphic stages when the animal already has numerous differentiated peripheral sensory cells. Moreover, the expression in these late larval stages was evenly distributed, so that no restriction to differentiating neural elements could be recognized, as is the case in other animals. In the *Drosophila* trunk sensory zones, for example, the expression of SoxNeuro demonstrates a solid full epithelium expression pattern only at the earlier stages of development. As the differentiation proceeds to sensory cells, sensory neurons, satellite cells, and covering epithelial cells, SoxNeuro expression gains a punctate pattern and gets restricted to pro-neurogenic cells only (Rizzo and Bejsovec, 2017). We do not observe a confinement of *LsSoxB1* expression to differentiating sensory elements in gastropods. This observation implies that mollusks exhibit distinctive features in the process of peripheral sensory cell differentiation.

In the deuterostome sea urchin, SoxB1 plays an important role in neurogenic differentiation (Feuda and Peter, 2022). Its expression covers the entire ectoderm at the time of larval neurogenesis, but is not confined to the proneurogenic zones only. SoxB2, in turn, shows a key function in the neurogenesis of the sea urchin and is mostly restricted to differentiating neuroblasts (Anishchenko et al., 2018). This interaction between SoxB family genes in echinoderms is strikingly similar to the expression patterns of *LsSoxB1* and *LsSoxB2* that we observe in gastropod *L. stagnalis*. Furthermore, *LsSoxB1*-positive cells in the invaginating endoderm in specific areas of the foregut and midgut, and cells in the oral cavity wall show expression exclusively at the mRNA level in the larvae of *L. stagnalis*. The same SoxB1 regulation at the translational level has been described in the endoderm of sea urchins (Wei et al., 2011), suggesting deep analogies between sea urchins and gastropod larvae. The origin of such parallelism is unclear and requires further investigation.

SoxB1 genes play a crucial role in maintaining a proliferative neurogenic state, extending beyond the neuroepithelium. Specifically, Sox2 is known for sustaining the stem cell status within proliferative zones in the central nervous system and retina throughout postnatal development and in adult vertebrates (Martínez-Cerdeño and Noctor, 2018). Secondary proliferative activity within the central nervous system is also observed in flatworms and some arthropods (Hartenstein and Stollewerk, 2015). In the nematode *C. elegans*, SoxB1 is not implicated in the differentiation of the majority of neurons and their epithelial progenitors. Instead, SoxB1 is required to maintain the developmental potential of blast cells generated in the embryo. These cells then divide and give rise to some differentiated neuronal cell types only post-embryonically (Holmberg et al., 2008). In gastropod, we observed that EdU incorporation reflecting DNA synthesis happens in *LsSoxB1*-positive cells within ganglia. A few of pH3-positive cells in the cortical layer of the cerebral and pedal ganglia also express *LsSoxB1*. This finding aligns with the fact that proliferation of proneurogenic cells within the ganglia in pulmonate gastropods described earlier (Anisimov and Kirsanova, 2002). In cephalopods, some divisions of SoxB1-positive cells have been described in cells along the migration pathway from the surface to the ganglia as well as within the ganglia themselves (Deryckere et al., 2021). Incorporation of EdU and phosphorylation of histone H3 in gastropod neurons are also anticipated to happen due to endomitosis events that accompany the process of neuron hypertrophy. Neurons in ganglia exhibit a gradual accumulation of ploidy throughout their lifespan, starting from early neurogenesis until the end of life, and can reach a ploidy of 16384C (Kirsanova and Anisimov, 2000, 2001; Anisimov, 2005). In *L. stagnalis*, we observed a lower count of *LsSoxB1* and pH3-positive cells compared to *LsSoxB1* and EdU-positive cells in the developing ganglia. Moreover, only a minimal number of cells within the ganglia, whether *LsSoxB1*-positive or *LsSoxB1*-negative, expressed pH3. These findings suggest that the endomitotic nuclear divisions accompanying neuronal polyploidy in *L. stagnalis* may not be closely associated with extensive histone H3 phosphorylation.

An intriguing result is the uniform distribution of cell divisions in the epithelium of *L. stagnalis*. We did not observe regions with concentrated proliferating cells in presumptive neurogenic zones at any of the analyzed developmental stages. This indicates that, in contrast to annelids, insects, and cephalopods, cell divisions (including presumptive neuronal precursors) in the neurogenic epithelium of gastropods probably occur over an extended period, and thus, cell divisions are spread in time without forming any clear proliferation-reach domain at any stage of development.

### 3.2 SoxB-family genes expression, early neurogenesis, and evolution of Mollusca

Data on early neurogenic events in gastropods are currently limited. Only a few morphological studies, focusing on the heterobranchs *Aplysia californica* and *Melibe leonina*, have described the processes of ectodermal cell delamination during the development of the central nervous system (CNS) ganglia (Kandel et al., 1980; Jacob, 1984; Page, 1992). In these works, the authors reported the development of cerebral ganglia from the ectoderm of cephalic plates and visceral, osphradial, and interstitial ganglia from the adjacent zones of visceropallial ectoderm. However, no systematic molecular-neurogenesis studies have been conducted on this subject.

SoxB1 expression at different developmental stages has been described for several mollusks, including gastropods. In the gastrula and trochophore of the polyplacophoran *Acanthochitona rubrolineata* (Huan et al., 2020), the SoxB1 expressing zone in the trunk was found to be expressed in spacious zones in the ventral trunk of the embryos. In the trochophore stage, all surface cells of the head except for the prototroch were SoxB1-positive. Such an expression pattern resembles what we observed in trochophores of *L. stagnalis*. However, in premetamorphic larvae of *A. rubrolineata*, the SoxB1-positive ectodermal zones noticeably diminished unlike in *L. stagnalis*. It is notable that a significant portion of SoxB1-positive cells is located subepithelially in *A. rubrolineata* similar to *L. stagnalis*.

SoxB1 expression during gastropod development has been previously described at the pregastrulation stages, in the gastrula and trochophore of the Patellogastropoda representatives *Patella vulgata* (Le Gouar et al., 2004) and *Lottia goshimai* (Huan et al., 2020; Tan et al., 2022). Notably, in both species at the early trochophore stage, broad expression of SoxB1 is observed in both ventral and dorsal parts of the head, which becomes limited to the ventral zone by the late trochophore stage. Additionally, SoxB1 expression is early extinguished in differentiating shell gland and prototroch cells. A similar pattern of SoxB1 expression was noted for *L. stagnalis*. However, in the trochophore of Patellogastropoda representatives, SoxB1 is expressed much less extensively. In the trunk, it is primarily restricted to the zone in the ventral part of the embryo. The posterior zone of the future foot in *L. goshimai* ceases to express SoxB1 by the end of the trochophore stage, whereas in *L. stagnalis*, the entire surface of the foot up to the late postmetamorphic stages is a zone of SoxB1 expression. It is worth noting that the trochophores of previously studied Patellogastropoda contain a much lower number of cells than *L. stagnalis* trochophore. In addition, the proportions between the parts of the embryonic body are different in patellogastropod and pulmonate gastropod *L. stagnalis* larvae. Specifically, broader zones of prototroch and shell gland cells are characteristic of patellogastropods. This may explain the observed differences in SoxB1 expression at early stages. Unfortunately, any data on the expression of SoxB1 in veliger or later stages including postmetamorphic animals is absent for Patellogastropoda. Tan et al. (2022) mentioned that zones of SoxB1 expression are adjacent to *Elav*-positive cells in subepithelial layers in *L. goshimai* trochophore. Together with the demonstration of subepithelial SoxB1 expression, it may be attributed to neurons. However, the lack of later stages with differentiated ganglia makes it difficult to clearly correlate the expression of SoxB1 with the developing nervous structures of Patellogastropoda.

The role of SoxB1 and SoxB2 in neurogenesis has been most thoroughly studied in cephalopods *Octopus vulgaris* and *Sepia officinalis* (Buresi et al., 2016; Focareta and Cole, 2016; Deryckere and Seuntjens, 2018; Deryckere et al., 2021; Duruz et al., 2023). The authors of all these papers mention that SoxB1 exhibits broad ectodermal expression at early neurogenic stages and further in development. SoxB1-positive ectodermal regions in cephalopods are accompanied by epithelial thickenings, which are hypothesized to give rise to neuronal precursors of the centralized brain. Single-cell transcriptomic data suggest the presence of SoxB1 both in neural migrative precursors together with the expression of Ascl and in some maturing neurons, supporting broad expression of SoxB1 in the migrating pro-neurogenic cells. Interestingly, we also observed numerous *LsSoxB1*-positive cells in a zone between epithelium and forming ganglia that may be referred to migrating neuroblasts in *L. stagnalis*. This observation allows us to speculate that migrating neuroblasts express SoxB1 in *L. stagnalis*. This assumption, however, needs further investigation.

One of the well-known conservative features of SoxB1 proteins is their ability to inhibit the activity of pro-neural factors and maintain pre-neural cells as undifferentiated precursors, retaining their ability to produce neuroblasts. Thus, the widespread expression of SoxB1 in cephalopods is usually attributed to their notably higher number of neurons in the brain compared to other lophotrochozoans. It is reasonable to assume that numerous neurons that make up the cephalopod brain arise from these expansive neurogenic zones. Unexpectedly, we found a similarly extensive expression of *LsSoxB1* in ectodermal areas in gastropod, although the number of neurons in their ganglia is considerably fewer than in cephalopods. This fact is consistent with the expression of SoxB2, which is mostly restricted to cells with neurogenic fate during neurogenesis. In vertebrates, the SoxB2-family gene Sox21 represses Sox3 (SoxB1 ortholog) and promotes terminal neural differentiation. Similarly, SoxB2 in mollusks may be a part of the mechanism that limits the number of central ganglion neurons generated during larval development.

On the other hand, Cephalopods are thought to have originated from a monoplacophoran or gastropod-like ancestor, thus sharing several developmental and anatomical features with gastropods (Shigeno et al., 2010). Thus, the expanded expression of SoxB1 in gastropod mollusks may constitute a component of a preadaptation complex that, having originated long ago, underlies the emergence of the sophisticated cephalopod brain and other distinctive traits during evolution. Further studies of molecular neurogenesis in gastropod larval development will shed light on the fascinating question of the evolution of the molluscan nervous system.

Another similarity between gastropods and cephalopods concerns the presence of SoxB2 in oral skeletogenic structures. In *L. stagnalis*, we found that *LsSoxB2* expression occurs in the epithelium of the developing radular sack. The presence of SoxB2 has also been documented in the epithelium responsible for the formation of skeletal oral structures in cephalopods (Focareta and Cole, 2016). This observation lends support to the idea of a common patterning of pharyngeal apparatus in gastropods and cephalopods. Taking into account the data on the non-neural pharyngeal expression of SoxB2 ortholog in the planarian *Schmidtea polychroa* (Monjo and Romero, 2015), and expression of SoxB2 in the developing mastax of monogonont rotifer *Brachionus manjavacas* (our unpublished data), suggests possible deeper conservation of SoxB2 in the development of the oral structures in spiralians. Moreover, we can infer more distant parallels to vertebrates, considering that Sox21 (one of the genes of the SoxB2 family) is expressed in the epithelium of developing teeth and plays a crucial role in the development of tooth enamel in mammals (Saito et al., 2020).

We observed an expanded and prolonged epithelial expression of SoxB1 in the larvae of *L. stagnalis*. Notably, the broad SoxB1 expression characteristic of the ectoderm in gastrulating embryos is also maintained in the epithelium of the post-metamorphic adult-like animal. This retention of embryonic features in later developmental stages suggests a phenomenon akin to neoteny, where an organism preserves characteristics of a younger stage as it progresses through development. In this context, neoteny refers to the protracted retention of embryonic traits in post-metamorphic stages, resulting in a heterochronic shift to the younger developmental stages compared to related animals. This concept parallels the idea that neoteny may contribute to enhanced cognitive abilities, as seen in human evolution with a prolonged period of heightened neuronal plasticity (Somel et al., 2009). The observed alteration in SoxB1 expression aligns with the concept of “transcriptional neoteny,” wherein gene expression in the adult organism mirrors that of an earlier developmental stage, accompanied by a shift in the regulation of corresponding developmental processes (Bakken et al., 2016). In various animals, including representatives of lophotrochozoans, the broad ectodermal expression of SoxB1 is typically limited to the pre-gastrulation and gastrulation stages, and becomes confined to neurogenic zones later in development (Okuda et al., 2010). Consequently, our finding that the gastropod retains a broad SoxB1 expression zone at late developmental stages can be considered a form of transcriptional neoteny. This phenomenon contributes to paedomorphic traits in gastropod mollusks, where complex of features can be interpreted as morphological expressions of heterochronic processes (Beklemishev, 1958a,b; Lindberg, 1988). It also aligns with the general neoteny hypothesis on the mollusks origin, previously discussed on the basis of purely morphological evidence (Garstang, 1928; Vagvolgyi, 1967).

### 3.3 Conclusion and future directions

In conclusion, our study unveils a nuanced and dynamic expression pattern of SoxB-family genes in the gastropod *L. stagnalis*, offering insights into the potential role of SoxB1 and SoxB2 in neurogenesis and morphogenesis in gastropods. The intriguing parallels with cephalopods and the unique expanded and prolonged SoxB1 expression pattern observed in gastropod *L. stagnalis* open up opportunities for further comparative and evolutionary studies to understand the molecular basis of neural development in mollusks. As we delve deeper into the intricacies of SoxB gene expression and its implications, we anticipate that this research will contribute to the broader field of evolutionary developmental biology.

## 4 Materials and methods

### 4.1 Animal handling

The freshwater pond snail *Lymnaea stagnalis* (*L. stagnalis*) is a pulmonate gastropod mollusk. The laboratory population of *L. stagnalis* at the Institute of Developmental Biology RAS originated from Vrije Universiteit, Amsterdam, in 1994. Mature snails were maintained under stable conditions (22–23°C, 16–8 h light–dark cycle) and provided with lettuce *ad libitum*. Egg masses were collected daily and examined under a dissecting microscope, with stages of embryonic development determined based on a comprehensive set of morphological characteristics following the method established by Meshcheryakov (1990).

### 4.2 The RNA extraction, RNA-Seq library preparation, and sequencing procedures

*L. stagnalis* embryos and postmetamorphic snails (st. 20–29) and adult nervous systems were utilized for total RNA isolation using the RNeasy Mini Kit (Qiagen, Hilden, Germany) in accordance with the manufacturer’s instructions.

The quality assessment of total RNA was performed using the Bioanalyzer 2,100 (Agilent, Santa Clara, CA, USA). The quantity and purity of RNA were determined on a NanoPhotometer (Implen). For library construction, 500 ng of total RNA with a RIN ≥7 was employed. The NEBNext® Poly(A) mRNA Magnetic Isolation Module and NEBNext® Ultra II™ Directional RNA Library Prep Kit for Illumina (New England Biolabs, Ipswich, MA, USA) were used based on the manufacturer’s instructions.

The quality verification of the libraries was conducted using the Bioanalyzer 2,100 (Agilent, Santa Clara, CA, USA), and the yield was validated through qPCR. Subsequently, the libraries were subjected to sequencing on HiSeq2500 (Illumina, San Diego, CA, USA) with pair-end 126 bp readings for transcriptome assembly.

### 4.3 *De novo* transcriptome assembly and analysis

Raw transcriptome assembly was carried out using the Trinity assembler (v 2.5.1) based on 5 paired-end libraries with 126 + 126 bp reads. The resulting set of transcripts underwent completeness analysis using the Busco software (v 2.0), utilizing the core Metazoa proteins dataset (*n* = 978).

To address the issue of overabundance inherent in raw transcriptome assemblies (characterized by a substantial number of duplicated transcripts), we implemented expression- and length-based filtration. Additionally, only the longest isoform for each Trinity-derived ‘gene’ was retained.

Abundance estimation involved the use of bowtie-2 for mapping and RSEM for calculating expression values. Subsequently, structural and functional annotation of the transcriptome was performed using Transdecoder (v 5.0.2) and Trinotate (v 3.1.1) tools.

### 4.4 Phylogenetic analysis

Sox protein sequences were identified through keyword searches in the NCBI GenBank^1^ (see Supplementary Table S1 for sequences accession numbers). Coding sequences for *L. stagnalis* were gathered by performing BLAST on molluskan and other lophotrochozoan HMG domains of Sox-family sequences against the *L. stagnalis* partial transcriptome using BLAST+ v. 2.11 software (Camacho et al., 2009). Putative Sox sequences identified via BLAST hits were translated using the Expasy Translate Tool.^2^ The HMG-containing open reading frames identified in the *L. stagnalis* coding sequences were then subjected to protein alignment using AliView v. 1.27 (Larsson, 2014) and the MAFFT multiple sequence alignment method (Katoh and Standley, 2013). Sequences missing part of the HMG domain were excluded from the analysis. The resulting alignment was utilized to calculate the phylogeny in IQ-tree v. 1.6.12 (Trifinopoulos et al., 2016), with Tcf-family proteins serving as an outgroup (Focareta and Cole, 2016). The LG + G4 phylogenetic evolution model was determined using IQ-tree protein Model Finder (Kalyaanamoorthy et al., 2017). Phylogenetic trees were constructed using IQ-tree with ultrafast bootstrapping (Nguyen et al., 2015). Tree visualization was performed using iTOL v. 6.8.1 (Ciccarelli et al., 2006).

### 4.5 HCR fluorescent *in situ* hybridization

The HCR probe pools for the fluorescent *in situ* mRNA visualization of *LsSoxB1* and *LsSoxB2* were meticulously generated using the modified HCR 3.0 *in situ* probe generator (Kuehn et al., 2022). To ensure optimal performance, the probe design incorporated filtration against stable secondary structures. Probes were synthesized in abundance, and potential off-target hybridization was rigorously screened using BLAST+. DNA pools, sourced from Synbio, Inc. (see Supplementary Table S2 for probe sets sequences), were dissolved in Tris-EDTA prepared with DEPC-treated DNase/RNase-Free MilliQ water. HCR amplifiers B1 with AlexaFluor 647 as fluorophore were procured from Molecular Instruments, Inc. The specificity of the HCR reaction was meticulously validated through probe-negative staining.

Whole *L. stagnalis* embryos were carefully extracted from the egg capsules and fixed in 4% paraformaldehyde for 2 h at room temperature. Subsequently, the samples underwent three washes with phosphate buffer (PBS) and were gradually dehydrated and stored in 100% methanol at −20°C. Preceding the HCR *in situ* hybridization (ISH) experiments, the samples were rehydrated in PBS through 10-min steps. The Molecular Instruments HCR ISH protocol designed for whole-mount sea urchin embryos (Choi et al., 2018) with minor modifications was applied. Post HCR-ISH, selected samples underwent cryosectioning and were labeled with antibodies, as detailed below. The whole mount preparations were immersed in 2,2′-thiodiethanol and prepared for confocal scanning microscopy.

### 4.6 Western blot analysis

For Western blot analysis, *L. stagnalis* larvae at the veliger stage (st. 22) and the body part without the shell and visceral complex of postmetamorphic snails (st. 29) were utilized. The *L. stagnalis* embryos were carefully removed from the eggs and washed gently in phosphate buffer saline to remove the egg mucus (PBS, pH 7.4). Tissue samples were promptly sonicated at 4°C in RIPA buffer (150 mM NaCl, 1.0% NP40, 0.5% sodium deoxycholate, 0.3% SDS, 50 mM Tris, pH 8.0) and then centrifuged at 12,000 g for 30 min at 4°C. Supernatants were employed for subsequent investigations. At least three technical replicates were performed for each developmental stage examined. Protein concentrations were determined using the BCA Protein Quantification Kit (Abcam, Cambridge, UK, ab102536) following the manufacturer’s instructions. The cleared homogenates were boiled for 5 min with β-mercaptoethanol. Polyacrylamide gels (10%) were loaded with samples (30 μg of protein/well), and electrophoresis was carried out for 30 min at 100 V and 90 min at 160 V in Tris/glycine/SDS running buffer. Proteins were then transferred to a nitrocellulose membrane (75 min at 80 V in a transfer buffer containing 0.3% Tris, 1.44% glycine, and 30% methanol). To confirm the success of the transfer, the membranes were stained with Ponseau S solution. Nonspecific binding was blocked by a 1-h incubation of the membrane in blocking buffer (TBS-T, 5% powdered milk), and membranes were subsequently incubated overnight at 4°C in blocking buffer with anti-Sox2 antibodies (Abcam, Cambridge, UK, ab97959, polyclonal, rabbit, 1:2000). Following several washes in TNT buffer, the membranes were incubated with anti-rabbit peroxidase-conjugated IgG (Jackson Immunoresearch, Cambridge, UK, 111–035-144, goat, 1:5000) for 2 h at room temperature. After the final washing in the TNT buffer, the membranes were revealed using the ECL detection system (Amersham Biosciences, UK, RPN2108).

### 4.7 Whole mount and cryosections immunostaining

*L. stagnalis* embryos at various developmental stages were extracted from the eggs and thoroughly washed in PBS. Subsequently, the samples underwent a 3-h fixation in 4% paraformaldehyde in PBS, followed by additional PBS washes. For the preparation of cryostat sections, selected samples were immersed in 20% sucrose in PBS for 24 h at 4°C and subsequently frozen at −40°C. Sections with a thickness of 20 μm were generated using the Leica CM1950 cryostat (Leica, Germany) and affixed to glass slides.

Immunolabeling procedures were consistent for both whole-mount preparations and cryostat sections, as well as for certain samples after HCR ISH and EdU incorporation. Preparations were initially washed in PBS and then incubated for 1.5 h at room temperature in 1% bovine serum albumin in PBS. Subsequently, preparations were exposed to various combinations of antibodies: anti-mouse Sox2 antibody (Abcam, Cambridge, UK, ab97959, polyclonal, rabbit, dilution 1:1000), anti-α-tubulin antibody (Sigma-Aldrich, Munich, Germany, T-6793, monoclonal, mouse, dilution 1:2000), and anti-FMRFamide antibody (Immunostar, Hudson, USA, 20091, polyclonal, rabbit, dilution 1:1000), all diluted in PBS containing 0.1% Triton-X100 and 0.1% bovine serum albumin, overnight at 4°C. After the antibody incubation, the preparations underwent a washing step and were then incubated in a mixture of secondary antibodies: anti-rabbit Alexa 488-conjugated IgG (Invitrogen, Waltham, USA; A-11008, goat, 1:700), anti-rabbit Alexa 555-conjugated IgG (Invitrogen, Waltham, USA; A-21428, goat, 1:700), and anti-mouse Alexa 633-conjugated IgG (Invitrogen, Waltham, USA; A-21050, goat, 1:700), all diluted in PBS containing 0.1% Triton-X100 and 0.1% bovine serum albumin, for 2 h at room temperature. Following a final washing step, nuclei were stained with DAPI and washed in PBS again. Whole-mount preparations were immersed in 90% glycerol and then mounted on slides, while cryosections were enclosed in a hydrophilic medium Mowiol (Sigma-Aldrich, Munich, Germany, 81,381).

### 4.8 Cell proliferation assays

Cell proliferation assays were conducted using 5-Ethynyl-2′-deoxyuridine (EdU; ThermoFisher Cat# C10337), a thymidine analog, and a rat antibody specific to phosphorylated histone H3 (Sigma-Aldrich, H9908). *L. stagnalis* larvae at various developmental stages, obtained from the eggs, were incubated in 200 μM EdU diluted in Lymnaea saline solution (prepared according to Meshcheryakov, 1990) for durations of 5 and 30 min. Afterward, larvae underwent two washes in Lymnaea saline and were fixed in 4% paraformaldehyde for 3 h. Some samples then underwent HCR ISH or antibody staining, using previously described techniques. Visualization of EdU incorporation was achieved using the Click-iT EdU Alexa Fluor 488 Imaging kit (ThermoFisher Cat# C10337). Immunolabeling with a rat anti-phospho-histone H3 antibody (polyclonal rat antibody against phosphorylated Ser28 histone H3, Sigma-Aldrich, H9908) was performed according to the outlined procedures, and staining was detected using anti-rat Alexa 555-conjugated IgG (ThermoFisher, Catalog # A-21434). Cell counting was conducted using the co-localization function and semi-automatic spot counting with Bitplane Imaris software. The unpaired t-test, following the F-test to confirm the equality of variances, was utilized to assess differences in means.

### 4.9 Microscopy and image proceeding

Preparations were examined utilizing a Zeiss LSM-880 confocal microscope (Carl Zeiss, Jena, Germany) with the application of appropriate wavelength-filter configurations. Subsequent to image acquisition, processing, and analysis of confocal images were performed using ZEN software (Carl Zeiss, Jena, Germany) and FIJI software.^3^

## Data availability statement

The datasets presented in this study can be found in online repositories. The names of the repository/repositories and accession number(s) can be found at: https://www.ncbi.nlm.nih.gov/genbank/, OR853093, OR853091, OR853092, and OR853094.

## Ethics statement

The manuscript presents research on animals that do not require ethical approval for their study.

## Author contributions

AK: Conceptualization, Investigation, Writing – original draft, Writing – review & editing. AF: Investigation, Writing – review & editing. MA: Investigation, Writing – review & editing. GG: Investigation, Writing – review & editing. OK: Data curation, Writing – review & editing. SV: Resources, Writing – review & editing. ES: Funding acquisition, Resources, Writing – review & editing. EI: Conceptualization, Data curation, Formal analysis, Investigation, Methodology, Project administration, Supervision, Writing – original draft, Writing – review & editing. EV: Conceptualization, Funding acquisition, Investigation, Project administration, Resources, Supervision, Writing – original draft, Writing – review & editing.

## Footnotes

^1^ https://www.ncbi.nlm.nih.gov/genbank/

^2^ https://web.expasy.org/translate/

^3^ http://fiji.sc/Fiji
