# Supplementary figures and images for: Expanded expression of pro-neurogenic factor SoxB1 during larval development of gastropod Lymnaea stagnalis suggests preadaptation to prolonged neurogenesis in Mollusca
Source: Front Neurosci. 2024 Apr 4;18:1346610. doi: 10.3389/fnins.2024.1346610 (PMC11024475; doi:10.3389/fnins.2024.1346610)

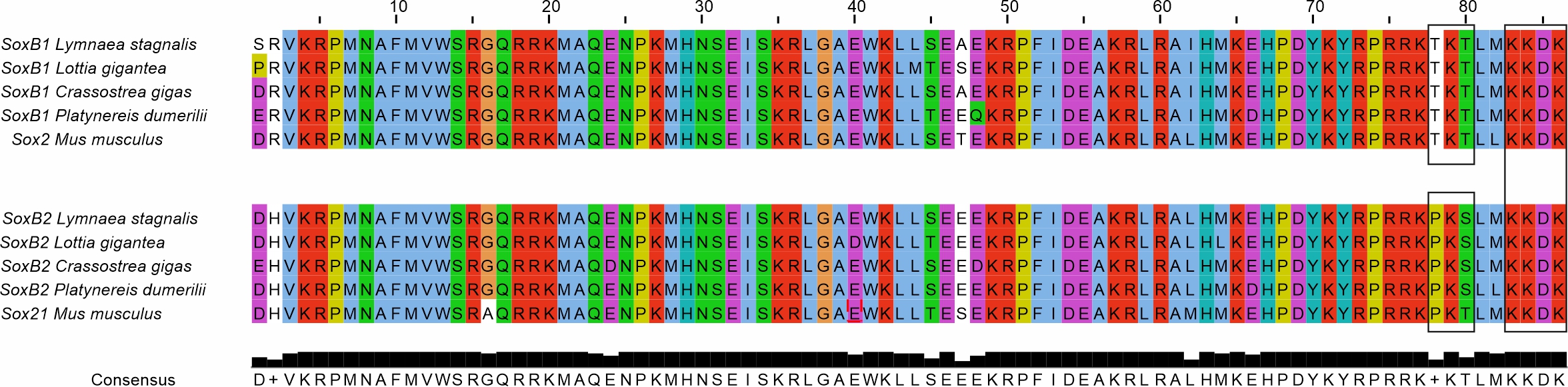

Supplement: Supplementary file 1 [file Image_1.JPEG]
